# DLL4 and VCAM1 enhance the emergence of T cell–competent hematopoietic progenitors from human pluripotent stem cells

**DOI:** 10.1126/sciadv.abn5522

**Published:** 2022-08-24

**Authors:** Yale S. Michaels, John M. Edgar, Matthew C. Major, Elizabeth L. Castle, Carla Zimmerman, Ting Yin, Andrew Hagner, Charles Lau, Han Hsuan Hsu, M. Iliana Ibañez-Rios, Lauren J. Durland, David J. H. F. Knapp, Peter W. Zandstra

**Affiliations:** ^1^ School of Biomedical Engineering, University of British Columbia, Vancouver, British Columbia V6T 1Z3, Canada.; ^2^ Institute of Biomedical Engineering, University of Toronto, Toronto, Ontario M5S 3G9, Canada.; ^3^ Institut de recherche en immunologie et en cancérologie and Département de pathologie et biologie cellulaire, Université de Montréal, Montreal, QC H3T 1J4, Canada.; ^4^ Department of Laboratory Medicine and Pathology, Mayo Clinic, Rochester, MN, USA.; ^5^ Michael Smith Laboratories, University of British Columbia, Vancouver, British Columbia V6T 1Z4, Canada.

## Abstract

T cells show tremendous efficacy as cellular therapeutics. However, obtaining primary T cells from human donors is expensive and variable. Pluripotent stem cells (PSCs) have the potential to provide a renewable source of T cells, but differentiating PSCs into hematopoietic progenitors with T cell potential remains an important challenge. Here, we report an efficient serum- and feeder-free system for differentiating human PSCs into hematopoietic progenitors and T cells. This fully defined approach allowed us to study the impact of individual proteins on blood emergence and differentiation. Providing DLL4 and VCAM1 during the endothelial-to-hematopoietic transition enhanced downstream progenitor T cell output by ~80-fold. These two proteins synergized to activate notch signaling in nascent hematopoietic stem and progenitor cells, and VCAM1 additionally promoted an inflammatory transcriptional program. We also established optimized medium formulations that enabled efficient and chemically defined maturation of functional CD8αβ^+^, CD4^−^, CD3^+^, TCRαβ^+^ T cells with a diverse TCR repertoire.

## Teaser

Engineered notch signals enable clinically relevant production of blood progenitors and T cells from pluripotent stem cells.

## INTRODUCTION

T cells are potent therapeutic agents. Chimeric antigen receptor (CAR) and engineered T cell receptor (TCR) T cells have demonstrated robust clinical efficacy against cancer (*1*–*3*) and show promise for treating infection and immunological disorders, as well as preventing transplant rejection (*4*, *5*). However, sourcing T cells from individual donors is expensive, time consuming, and laborious (*6*). Interdonor variability and intradonor heterogeneity in CAR/TCR transduction make personalized T cell therapies difficult to manufacture and quality control (*7*–*9*).

Human pluripotent stem cells (hPSCs) offer an attractive solution to these manufacturing challenges (*10*). hPSCs have the capacity for unlimited self-renewal. They can be engineered, clonally selected, and screened for quality control before expansion, enabling scalable production of a homogeneous product (*11*). Developing efficient processes for differentiating hPSCs into T cells will enable cost-effective and consistent cell therapy manufacturing, ultimately leading to more efficacious products (*12*–*17*).

A major bottleneck on the path to T cell production is to efficiently differentiate hPSCs into definitive hematopoietic progenitors with T cell potential (*18*–*21*). Definitive hematopoietic stem cells (HSCs) arise from a cell type known as hemogenic endothelium in a process called the endothelial-to-hematopoietic transition (EHT) (*22*, *23*). Previous methods for producing T lineage–competent hematopoietic stem and progenitor cells (HSPCs) from hPSCs have used immortalized stromal cell lines and serum or undefined cellular extracts to support EHT and T cell differentiation (*12*, *14*, *15*, *17*, *20*, *24*). These undefined components obfuscate the signaling interactions that drive development, making key parameters difficult to identify and optimize. The use of animal-derived sera, feeder cells, and undefined extracts also causes lot-to-lot variability and limits translation to the clinic (*25*).

Iriguchi *et al.* (*13*) recently developed a feeder-free protocol for differentiating induced pluripotent stem cells (iPSCs) into T cells. Using iPSCs that already harbored rearranged TCRs, either by reprogramming from a T cell clone or through TCR transduction, authors were able to generate CD8^+^, CD4^−^, CD3^+^, TCRαβ^+^ T cells. Despite this important advance, iPSCs without a prerearranged TCR failed to develop into TCRαβ^+^ T cells, suggesting that their process failed to capture key aspects of conventional T cell development (*13*).

In another recent study, Trotman-Grant *et al.* (*16*) succeeded in generating CD4^+^, CD8^+^, CD3^+^, TCRαβ^+^ T cell progenitors from iPSCs without the need to supply a rearranged TCR, achieving an important milestone. However, in this protocol, the efficiency of converting CD34^+^ cells to T cell progenitors cells was low (estimated by the authors to be on the order of 1/1000), consistent with a bottleneck at the stage of generating T lymphoid–competent hematopoietic cells (*16*). The authors first generated CD34^+^ cells in a mesoderm/hematopoietic differentiation, which they subsequently seeded into a T cell differentiation culture (*16*). The protocol that they used to generate CD34^+^ cells has been reported to primarily produce hemogenic endothelial (HE) cells, the developmental precursor of HSC (*21*). These cells may not be at the appropriate developmental stage to begin T cell differentiation.

Here, we hypothesized that adding an EHT stage to the differentiation process would allow us to convert cells to T lymphoid–competent hematopoietic progenitors at the appropriate developmental stage to progress into T cell differentiation conditions. We aimed to use recombinant proteins, rather than stromal cell lines, serum, or undefined extracts, to enable better control over the EHT process and to gain insight into the biological requirements for T lymphoid–competent hematopoiesis. The developmental niches where hematopoietic cells emerge are complex three-dimensional (3D) environments comprising multiple cell types and a milieu of paracrine, autocrine, and juxtracrine signals that have not been completely mapped in humans (*26*). Rather than attempting to recapitulate this complexity in vitro, we sought to establish a simplified minimal system where the relative contribution of individual factors can easily be manipulated and tested, and where protocol improvements can be readily translated toward clinical development.

Prior work suggests that notch signaling during EHT is required to produce T lymphoid–competent hematopoietic cells (*24*, *27*). This motivated us to test the potent notch ligand DLL4 in our culture system. Transcriptional profiling in mouse revealed that the cell adhesion molecule VCAM1 is highly enriched in cells of aorta-gonad-mesonephros (AGM) capable of supporting definitive hematopoiesis (*28*). Our laboratory has shown that VCAM1 is able to synergize with DLL4 to enhance notch signaling in a different developmental context (*29*). Consequently, in this study, we also tested the impact of VCAM1 during EHT. We show that DLL4 and VCAM1 interact to markedly improve production of hematopoietic progenitors with T cell potential from hPSCs. Collectively, the addition of these two proteins during EHT increased downstream T cell progenitor output by more than 80-fold.

The chemically defined differentiation method we developed here allowed us to probe the impact of each immobilized protein on hematopoietic lineage output and transcriptional programs. This analysis revealed that VCAM1 promotes an inflammatory transcriptional program in emergent HSPCs. Last, this method enabled the production of not only T cell progenitors but also mature CD8αβ^+^, CD4^−^, CD3^+^, CD62L^+^, TCRαβ^+^ T cells and allowed us to study the impact of cytokine exposure dynamics on T cell maturation. Our platform provides a customizable environment capable of revealing key signaling requirements for blood emergence and T cell development. This new method will contribute to robust, clinically compatible manufacturing of PSC-derived T cell therapies.

## RESULTS

### DLL4 and VCAM1 enhance the emergence of T cell competent hematopoietic progenitors from hPSCs

**Fig. 1.**
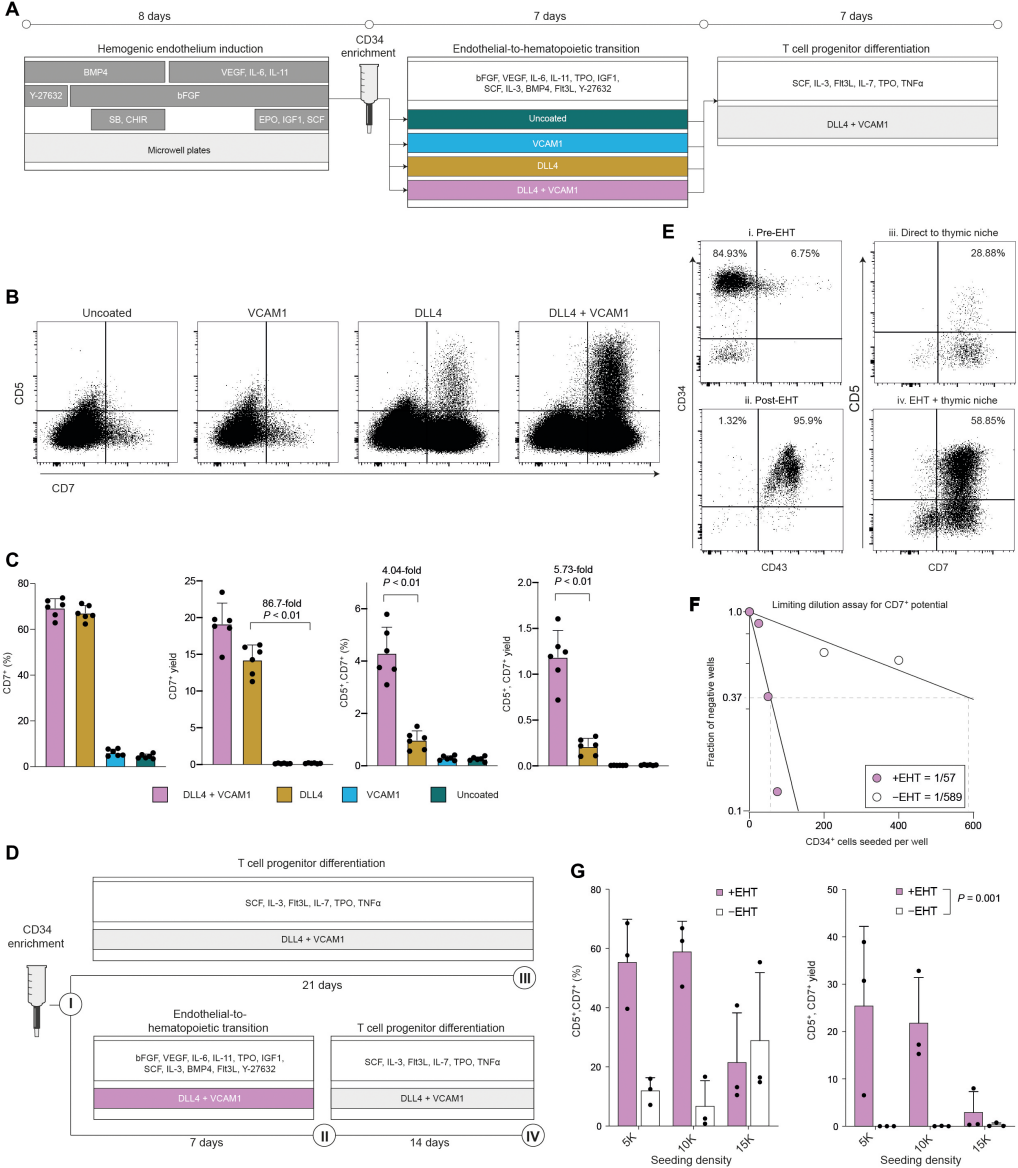
The presence of DLL4 and VCAM1 during the endothelial to hematopoietic transition supports development of HSPC with robust T cell potential. (**A**) Schematic overview of chemically defined platform for producing multipotent hematopoietic progenitors and T cell progenitors from pluripotent stem cells. (**B**) Flow cytometry analysis of progenitor T cell output after transitioning cells from 7 days in each EHT coating condition into a common defined thymic niche for an additional 7 days. (**C**) Quantification of the frequency and yield of CD7^+^ lymphoid progenitors and CD7^+^, CD5^+^ T cell progenitors after 7 days in the thymic niche (mean ± SD, *n* = 6). Indicated *P* values reflect result of Mann-Whitney test. (**D**) Experimental design to assess the effect of adding or omitting the EHT culture phase before transferring cells into the thymic niche. (**E**) Immunophenotype of cells generated with or without the EHT culture stage. Numerals in (E) correspond to the schematic in (D). (**F**) Results of limiting dilution analysis to assess the frequency of cells with CD7^+^ NK/T lymphoid potential within the CD34^+^ population with or without an EHT step. Indicated cell numbers were seeded in 96-well plates and cultured for a total of 14 days (either 7 days EHT + 7 days pro-T differentiation medium or 14 days pro-T differentiation medium). Wells were scored as positive if they contained >25 viable CD7^+^ cells. Frequencies were modeled as the number of cells required to achieve a failure rate of 0.37. (**G**) Quantification of the yield and frequency of T cell progenitors generated with or without an EHT culture phase. Indicated *P* value is for the effect of EHT on CD5^+^, CD7^+^ yield, two-way ANOVA.

We set out to understand the minimal signaling requirements for generating hematopoietic progenitors with T cell potential from hPSCs. We first attempted to generate HE cells, the developmental precursors of HSCs, by aggregating hPSC into 3D structures by centrifugation in microwell plates (*30*). We subjected the aggregates to a step-wise series of medium formulations known to specify mesoderm, and subsequently definitive hemato-endothelial identity (*21*, *27*). We used small molecules to inhibit activin signaling and promote Wnt pathway activity during the early stage of the differentiation, as this has previously been shown to suppress the primitive hematopoietic program and promote the emergence of HE cells with high transcriptional similarity to the human AGM (*21*, *31*). We assayed the aggregates by flow cytometry and verified that the mesoderm generated in our differentiations lacked CD235a expression, a marker of cells fated toward a primitive/yolk sac identity (fig. S1) (*21*, *32*). After 8 days of differentiation, we dissociated the resulting aggregates and separated CD34^+^ cells to enrich for HE. We placed the CD34^+^ cells onto an uncoated tissue culture surface in medium formulated to promote EHT (Fig. 1A) (*33*). We observed a morphological transition from adherent, endothelial-like cells to nonadherent spherical cells (fig. S1).

Next, we assayed whether these nonadherent hematopoietic cells were capable of differentiating into T cell progenitors. We transferred the cells to plates coated with immobilized DLL4, a notch ligand capable of promoting T lineage specification, and VCAM1, a cell adhesion molecule that we have previously shown to enhance this process (Fig. 1A) (*29*). Although we cultured the cells in medium capable of generating CD5^+^, CD7^+^ T cell progenitors from cord blood stem cells, we detected almost no CD5^+^, CD7^+^ cells from hPSC-derived hematopoietic cells (Fig. 1, B and C). We observed very low numbers of CD7^+^ cells, an earlier and less committed progenitor (Fig. 1, B and C).

Multiple reports suggest that notch signaling during EHT promotes the emergence of HSPC with T lineage potential (*24*, *27*). Notably, OP9 stromal cells engineered to express DLL4 are able to support definitive hematopoiesis from arterial HE (*24*). We attempted to obviate the need for feeder cells by including immobilized recombinant DLL4 during the EHT culture phase (Fig. 1A). When we transferred the HSPC generated on DLL4 into conditions that support T cell development, we observed a marked improvement in progenitor T cell differentiation compared to HSPC generated on uncoated plates. The presence of DLL4 during EHT increased the median frequency of CD7^+^ cells generated after 7 days in downstream culture to 66.51% compared to 4.22% in the uncoated condition (*P* = 0.002, Mann-Whitney *U* test; Fig. 1C). DLL4 increased the yield of CD7^+^ cells produced per input CD34^+^ cell ~90-fold compared to the uncoated control (Fig. 1C). Encouragingly, DLL4 led to the emergence of a distinct population of the later-stage CD5^+^, CD7^+^ T cell progenitors, a population that was nearly absent from the uncoated condition (Fig. 1B). These data demonstrate that the addition of recombinant DLL4 during a chemically defined EHT phase markedly improves the production of hematopoietic cells with T lineage potential from hPSCs.

Next, we attempted to improve progenitor T cell output even further. We previously demonstrated that the cell adhesion protein VCAM1 can increase the magnitude of notch signaling imparted by immobilized DLL4 during in vitro T cell differentiation (*29*). We asked whether we could apply this principle to increase notch signaling during EHT and thus increase T cell potency.

When we supplied VCAM1 in combination with DLL4 during EHT, we observed a marked increase in downstream production of CD5^+^, CD7^+^ T cell progenitors (Fig. 1B). VCAM1 and DLL4 together increased both the frequency (4.04-fold, *P* = 0.0022, Mann-Whitney test) and yield per-input CD34^+^ cell (5.73-fold, *P* = 0.0022, Mann-Whitney test) of CD5^+^, CD7^+^ cells compared to DLL4 alone (Fig. 1C). VCAM1 alone did not substantially alter progenitor T cell production compare to the uncoated control (Fig. 1, B and C), suggesting that it acts cooperatively with DLL4 to enhance the differentiation. This effect cannot be attributed to an increase in the number of nonadherent hematopoietic cells produced during EHT, as this number did not differ substantially between coating conditions (fig. S1).

We tested whether T cell progenitor yields are improved by this EHT culture phase, compared to the method presented by Trotman-Grant *et al.* (*16*), where CD34-enriched cells harvested from aggregates are placed directly into conditions that support T cell differentiation (Fig. 1D). In agreement with previous reports, we found that >90% of CD34^+^ cells harvested from the day 8 aggregates generated in our protocol were CD43^−^, indicating that they have not yet established a hematopoietic identity (Fig. 1E) (*21*). In contrast, >95% of the nonadherent cells harvested after EHT coexpressed CD34 and CD43 (Fig. 1E). Consistent with this transition to hematopoietic identity, the EHT phase improved the yield of CD5^+^, CD7^+^ T cell progenitors by 70-fold compared to seeding CD34^+^ cells directly into T cell differentiation conditions (Fig. 1, E and G). The EHT phase significantly improved T cell progenitor yield across multiple cell seeding densities [Fig. 1G; *P* = 0.001, two-way analysis of variance (ANOVA)].

To assess whether the addition of the EHT phase increased the frequency of CD34^+^ cells that are able to give rise to CD7^+^, natural killer (NK)/T lymphoid progenitors, we performed a limiting dilution analysis (Fig. 1F). In the method reported by Trotman-Grant *et al.* (*16*), which lacks an explicit EHT stage, 1 of 1341 CD34^+^ cells were able to give rise to NK/T lymphoid progenitors. The frequency in our own no-EHT control was 1/589 (Fig. 1F). Notably, the addition of the EHT phase increased the frequency to 1/57, a 10-fold improvement over our internal no-EHT control and a >20-fold increase against the previously published results (Fig. 1F) (*16*). We conclude that an engineered signaling environment comprising recombinant DLL4 and VCAM1 and an appropriate chemically defined, serum-free medium is sufficient to support emergence of HSPC with robust T lineage potential.

### DLL4 shifts hPSC-derived hematopoietic cell fate outputs

The addition of immobilized DLL4 and VCAM1 during EHT markedly affected the ability of PSC-derived HSPC to differentiate toward the T lineage. We asked whether we could identify differences within the upstream HSPC population. First, we profiled the nonadherent cells that emerged during EHT using a comprehensive flow cytometry panel designed to identify definitive HSPC populations from fetal liver (FL) and umbilical cord blood (UCB) (*34*). PSC-derived HSPC from all four coating conditions contained distinct populations of CD45^+^, CD34^+^, CD38^−^, CD7^−^, CD10^−^, CD90^+^, CD45RA^−^, CD49F^+^ HSCs and CD45^+^, CD34^+^, CD38^−^, CD7^−^, CD10^−^, CD90^−^, CD45RA^−^, CD49F^−^ multipotent progenitors (MPPs) (fig. S2). Unexpectedly, the prevalence of phenotypic HSCs was elevated in the uncoated condition by ~2% (fig. S2). The addition of DLL4 increased the frequency of CD45^+^, CD90^−^, CD45RA^+^, CD7^+^ multi-lymphoid progenitors by approximately twofold independent of VCAM1 (fig. S2).

**Fig. 2.**
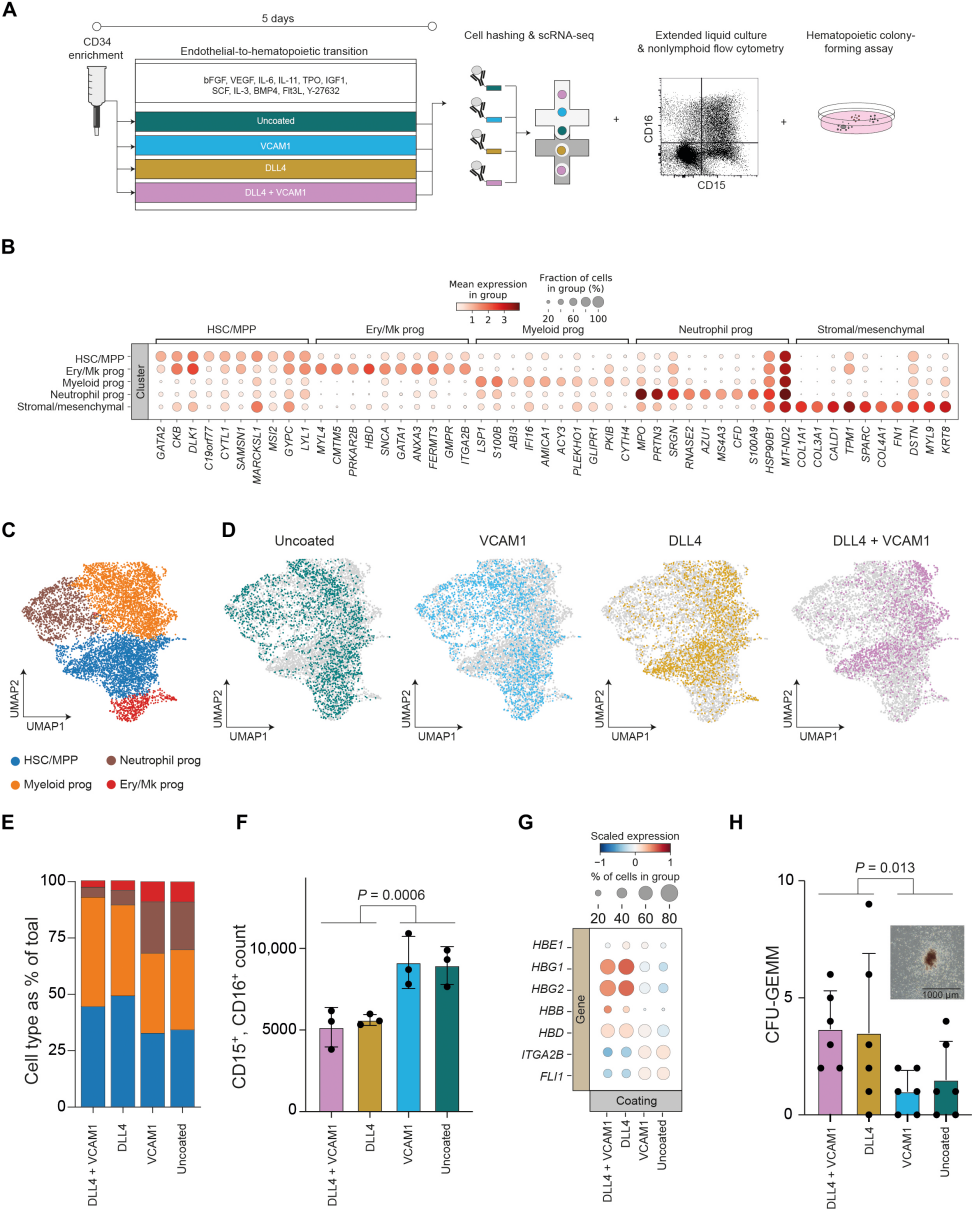
Engineered notch signaling during EHT reduces neutrophil differentiation and promotes definitive hematopoiesis. (**A**) Schematic overview of the experimental design used to test how the presence of DLL4 and VCAM1 during EHT affects the resulting HSPC. (**B**) We performed unsupervised Leiden clustering and identified five cell clusters. Clusters were annotated on the basis of differential gene expression, and the top 10 differentially expressed genes for each cluster are displayed. (**C**) UMAP projection of cells identified in scRNA-seq after quality control filtration and removal of stromal/mesenchymal cells. Cells are colored by unsupervised Leiden clusters. (**D**) UMAPs as in (C), colored by coating condition. (**E**) Proportion of each hematopoietic progenitor subtype identified in scRNA-seq data, quantified for each coating condition. (**F**) Flow cytometry quantification of neutrophil output from HSPC generated in each EHT coating condition following extended 7-day liquid culture in myeloid and erythroid supportive medium. Indicated *P* value is for the effect of EHT on CD5^+^, CD7^+^ yield, two-way ANOVA. (**G**) Comparison by coating condition of expression of hemoglobin genes and megakaryocyte (Mk)–associated transcription factors within the Mk/erythroid progenitor cluster. Expression is scaled across all cells to a mean of 0 and unit variance. DLL4 significantly increases expression of *HBG1* (log_2_ fold change = 2.31, *P*_adjusted_ = 0.000031, Mann-Whitney *U* test) and *HBG2* (log_2_ fold change = 2.40, *P*_adjusted_ = 0.000069, Mann-Whitney *U* test). (**H**) CFU-GEMM quantification from HSPC generated in each EHT coating condition following 14-day culture in semisolid medium (mean ± SD, *n* = 6). Indicated *P* value is for the effect of EHT on CD5^+^, CD7^+^ yield, two-way ANOVA. Representative colony image is inset; scale bar, 1000 μm.

Next, to understand how DLL4 and VCAM1 altered the cellular identity of PSC-derived HSPC at higher resolution, we used droplet-based single-cell RNA sequencing (scRNA-seq) (Fig. 2A) (*35*). We compared nonadherent cells generated during EHT on uncoated plates, plates coated with DLL4 or VCAM1 alone, or plates coated with both proteins. Following doublet and dead-cell removal, we obtained 7589 cells, including at least 1500 from each of the four coating conditions. We performed unbiased clustering and differential gene expression analysis (Fig. 2, B and C, and fig. S3) (*36*). We detected a small population of stromal/mesenchymal cells that we excluded from downstream analysis (fig. S3). We annotated the remaining four clusters as subtypes of HSPC on the basis of differential gene expression: *GATA2*^+^*, CYTL1*^+^ HSCs/MPP, *LSP1*^+^, *ABI3*^+^, *IFI16*^+^ myeloid progenitors, *HBD*^+^, *ITGA2B*^+^ erythroid/megakaryocyte progenitors, and *SRGN*^+^, *MPO*^+^ neutrophil progenitors (Fig. 2B and fig. S3). Most cells expressed the hematopoietic gene *SPN*, while the endothelial gene *CDH5* was nearly absent, consistent with successful transition from an endothelial to hematopoietic transcriptional program (fig. S3). Although *CD34* expression varies across cells at the RNA level (fig. S3), the nearly ubiquitous cell surface expression of CD34 as measured by flow cytometry (Fig. 1E) motivated us to label these hematopoietic subpopulations as progenitors.

scRNA-seq revealed that coating conditions during EHT shifted the relative proportions of the resulting HSPC subtypes (Fig. 2, D and E). The presence of DLL4 caused an increase in the frequency of HSC/MPP, decreased the occurrence of erythroid/megakaryocyte progenitors, and strongly reduced the abundance of neutrophil progenitors compared to VCAM1 alone or the uncoated control (Fig. 2E). Upon extended liquid culture, we confirmed that DLL4 reduced the output of CD15^+^, CD16^+^ neutrophils 1.68-fold (Fig. 2F; *P* = 0.0006, two-way ANOVA).

Within the erythroid/megakaryocyte progenitor population, we observed that DLL4 reduced expression of the megakaryocyte transcription factors *ITGA2B* and *FLI1*, and increased expression of hemoglobin genes associated with erythroid specification (Fig. 2G). When we subclustered these cells, we observed an increase in the ratio of *KLF1*-expressing erythroid to *PLEK*-expressing megakaryocyte progenitors (*37*) in the presence of DLL4 (fig. S4). Extended liquid culture verified that DLL4 increased the ratio of CD235a^+^ erythroid cells to CD41^+^ megakaryocytes by 2.18-fold (fig. S5; *P* < 0.0001, two-way ANOVA).

Notch signaling during EHT in vitro has been reported to promote emergence of HSPC comparable to a later stage in human ontogeny as evidenced by a switch in globin gene expression from embryonic to fetal (*24*). We examined globin gene expression within the erythroid/megakaryocyte cluster (Fig. 2G). Upon addition of DLL4, we observed increased expression of the fetal globin genes *HBG1* (log_2_ fold change = 2.31, *P*_adjusted_ = 0.000031, Mann-Whitney *U* test) and *HBG2* (log_2_ fold change = 2.40, *P*_adjusted_ = 0.000069, Mann-Whitney *U* test) (Fig. 2G).

Colony-forming assays demonstrated that DLL4 increased the frequency of the multi-lineage granulocyte, erythrocyte, monocyte, megakaryocyte colony-forming unit (CFU-GEMM) by 2.9-fold (Fig. 2H; *P* = 0.013, two-way ANOVA), without significantly altering CFU-E and CFU-GM output (fig. S5; *P*_CFU-E_ = 0.065, *P*_CFU-GM_ = 0.91, two-way ANOVA). DLL4 drives a shift in HSPC cell composition, including a reduction in neutrophil output, higher levels of fetal and adult hemoglobin expression, and an increase in the frequency of multipotent CFU-GEMM. While VCAM1 substantially enhanced T cell progenitor differentiation, this protein did not substantially alter the proportions of HSPC subtypes. To understand how the presence of DLL4 and VCAM1 during EHT influences T lymphoid competency, we analyzed transcriptional changes within the HSC/MPP cluster, the cell type from which lymphoid progenitors arise.

### DLL4 and VCAM1 alter HSC/MPP gene expression programs and cooperatively activate notch signaling

**Fig. 3.**
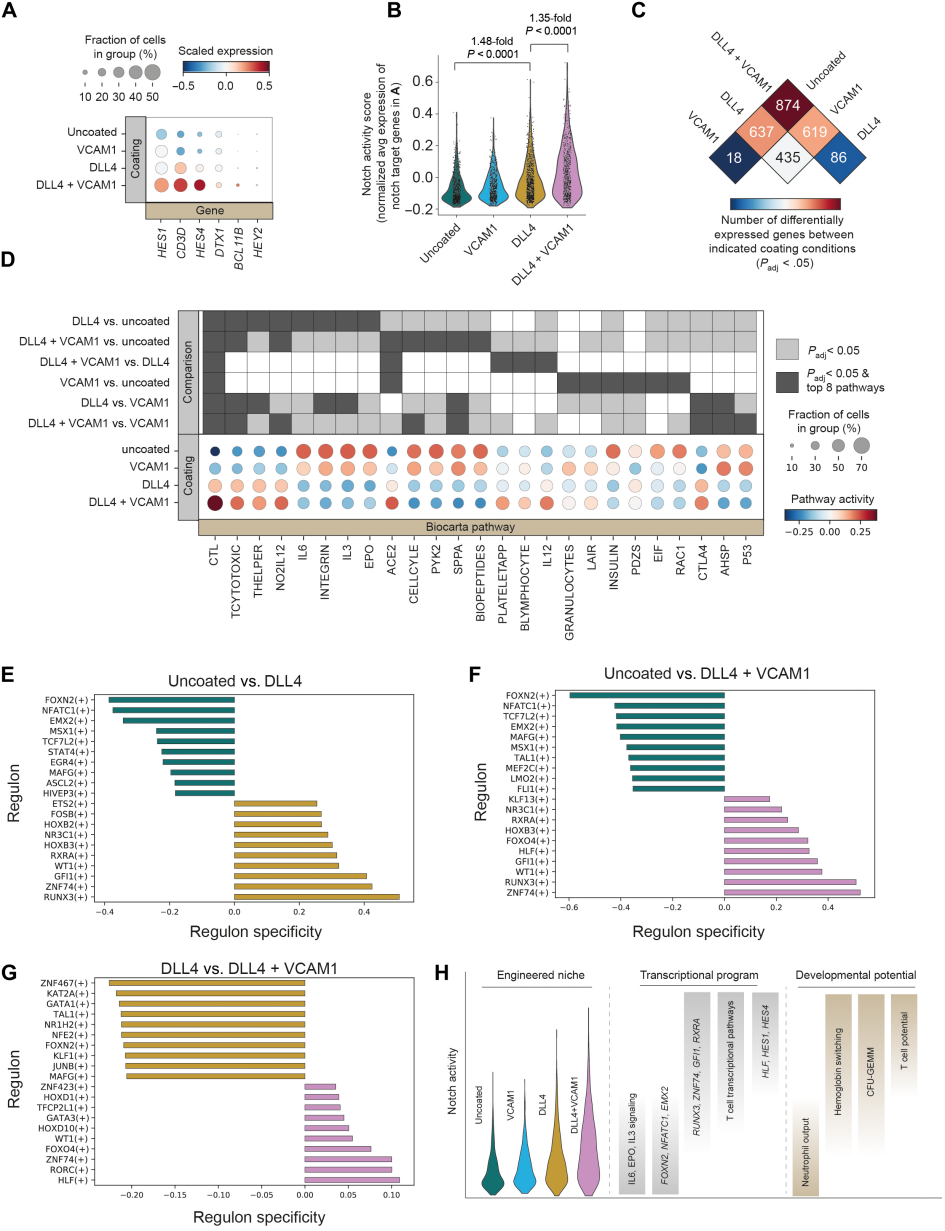
VCAM1 promotes an inflammatory program and cooperates with DLL4 to enhance notch signaling and hematopoietic gene expression in HSC/MPP. (**A**) Expression of known notch target genes was analyzed in cells within the HSC/MPP cluster from each coating condition. Expression of each gene scaled to a mean of 0 and unit variance within all HSC/MPP. (**B**) The average of the notch target genes shown in (A) was combined on a per-cell basis and normalized to the average of 50 randomly selected reference genes selected from the same expression bins to create a single-cell notch activity score. The notch activity score is reported for HSC/MPP from each coating condition. *P* values reflect two-way ANOVA, Tukey’s multiple comparison test. (**C**) Differential expression analysis (Wilcoxon rank sum) was performed on the HSC/MPP cells between each pair of coating conditions. Values indicate the number of significantly differentially expressed genes (*P*_*a*djusted_ < 0.05) identified in each pairwise comparison. (**D**) Differential pathway activity within HSC/MPP across coating conditions. Pairwise comparisons were performed between each coating condition and all others, and the top eight differentially active pathways by *z* score from each comparison are depicted. Box shading indicates which pathways are in the top eight of a given comparison and which comparisons have *P*_adjusted_ < 0.05. (**E** to **G**) Comparison of SCENIC regulon activity between coating conditions. The top 10 regulons specific to each coating condition are shown. Regulon specificity is calculated as the Jensen-Shannon distance between conditions. (**H**) Model of the impact of coating conditions on notch signaling and associated notch dependence of transcriptional programs and cell fate decisions.

We examined how the engineered signaling environment affected the transcriptional identity of HSC/MPP produced during EHT. Expectedly, the presence of DLL4 increased the frequency and magnitude of expression of known downstream targets (*38*–*41*) of notch signaling (Fig. 3, A and B). In a separate developmental context, we previously showed that the cell adhesion molecule VCAM1 can enhance DLL4-mediated notch signaling (*29*). VCAM1 alone did not substantially alter expression of notch targets (Fig. 3B). In combination with DLL4, VCAM1 markedly increased the mean expression of *HES1*, *CD3D*, *HES4*, *DTX1*, *BCL11B*, and *HEY2* compared to DLL4 alone (Fig. 3, A and B; 1.35-fold increase in notch activity score, *P* = 3.36 × 10^−12^, Mann-Whitney *U* test). During EHT, VCAM synergizes with DLL4 to promote high levels of notch activity in HSC/MPP (two-way ANOVA, interaction *P* < 0.0001).

Next, we undertook an unbiased exploration of the impact of coating conditions on MPP/HSC gene expression (Fig. 3C and fig. S6). The greatest number of significantly differentially expressed genes was observed between the DLL4 + VCAM1 condition and the uncoated condition (874 genes; Fig. 3C). In contrast, only 18 genes significantly differed in expression between the VCAM1 and uncoated EHT conditions (Fig. 3C). In addition to increasing notch signaling, differential expression analyses revealed that VCAM1 also altered expression of *EVL* and *FERMT3*, genes involved in cell adhesion and cytoskeletal polymerization (*42*, *43*), and increased activity of the interferon (IFN)–induced genes *IFI6* and *IRF1* as well as members of IFN-induced immunoproteosome, *PSME2* and *PSME1* (fig. S6). Changes in proinflammatory, IFN-responsive genes were observed, both when comparing VCAM1 to the uncoated control and when comparing VCAM1 and DLL4 to DLL4 alone (fig. S6).

Pathways associated with lymphoid and T cell identity were among the most strongly activated by DLL4 (Fig. 3D). Cytotoxic T lymphocyte (CTL), TCYTOTOXIC, and THELPER were all highly up-regulated by DLL4, and these effects were magnified by the addition of VCAM1 (Fig. 3D). DLL4 reduced activity of cell cycle and P53 pathways and increased activity of the T cell inhibitory pathway CTLA4. These alterations in transcriptional state are consistent with a model whereby notch signaling promotes emergence of an HSC/MPP population that is primed to undergo TCR-mediated selection during T cell differentiation.

SCENIC analysis (*44*) revealed regulons (transcription factors and their downstream targets) that were specifically up-regulated in each engineered signaling environment (Fig. 3, E to G). Nearly all of the regulons that were most strongly activated by DLL4 have a known role in blood emergence and T cell differentiation in human or mouse (Fig. 3E). *RUNX3* contributes to HSC maintenance and T cell differentiation (*45*, *46*). *GFI1* and *FOSB* are important drivers of endothelial to hematopoietic transition (*47*, *48*). The homeobox gene *HOXB3* is expressed in both uncommitted HSCs and during T cell development (*49*). *WT1* is important for survival and maintenance of HSC/MPP and down-regulated in differentiated progeny (*50*, *51*). The DLL4-mediated increase in *RXRA* regulon activity merits future investigation given the complex role of retinoic acid signaling during the emergence of HSCs from hPSCs (*52*–*54*). *ZNF74* was also among the regulons most strongly up-regulated by DLL4 and has no previously documented role in hematopoiesis (Fig. 3, E and F).

Notably, when added alongside DLL4, VCAM1 increased *HLF* and *GATA3* regulon activity (Fig. 3G). *HLF* expression is highly correlated with definitive HSC identity in human cord blood, FL, peripheral blood, and bone marrow (*55*). In addition to its appreciated role in human T cell development, *Gata3* helps maintain HSC identity in mice (*56*).

Collectively, these data demonstrate that introducing DLL4 and VCAM1 during EHT positively shifts the distribution of notch pathway activity in the resulting HSC/MPP (Fig. 3, B and H). This shift in notch activity is associated with marked changes in transcription factor and pathway activity and alters cell fate distribution, with intermediate levels of notch activity increasing definitive erythroid output at the expense of megakaryocytes and suppressing neutrophil output and high notch promoting T cell potential (Fig. 3H).

### Mapping PSC-derived HSPC to primary definitive hematopoietic cells

To validate our key findings, and to understand how the HSPCs generated in our in vitro differentiation protocol compare to primary hematopoietic cells, we integrated our scRNA-seq data with a published analysis of the human FL. We used a global anchor-based integration strategy (*57*) to compare the PSC-derived HSPC in our study with FL cell types from Popescu *et al.* (*58*). We found that cells in our PSC-derived HSC/MPP cluster were most similar to the annotated HSC/MPP in the primary FL dataset (fig. S7) (*58*). PSC-derived HSC/MPP also shows similarity to a population of megakaryocyte–erythroid–mast cell progenitors (MEMPs) in the FL dataset (fig. S7).

We asked whether coating conditions during EHT affected the similarity of the resulting PSC-derived HSPC to different FL cell types (*58*). We found that the HSC/MPP generated from PSCs in the presence of DLL4 + VCAM1 had the highest correspondence with primary HSC/MPP when compared to the other coating conditions (fig. S7). DLL4 + VCAM1 also led to the highest score for early lymphoid/T lymphocyte progenitors, followed by DLL4 alone. Consistent with our own unsupervised clustering and extended liquid culture experiments, DLL4 coating increased the FL early erythroid score and decreased the scores for both FL-megakaryocyte and FL-neutrophil-myeloid progenitors (fig. S7).

Next, we used the anchor-based integration approach (*57*) to classify the cells in our dataset into the cell types labeled in the Popescu *et al.* (*58*) FL dataset. Using these FL labels, we calculated the frequency of each cell type across coating conditions (fig. S7). This analysis strongly supports the main conclusions of our own unsupervised clustering, colony-forming assays, and extended liquid culture. Specifically, using the FL labels, we confirm that DLL4 increases the abundance of HSC/MPP and erythroid cells while decreasing the frequency of megakaryocytes and neutrophils (fig. S7).

To further validate this comparison, and to place our PSC-derived cells along the continuum of human blood development with increased resolution, we also integrated (*57*) our dataset with a recently published analysis of human HSC emergence (*59*). This reference dataset contains primary cells from multiple time points and anatomical locations including the FL and AGM (*59*). This global integration analysis confirmed that our PSC-derived cells show strong transcriptional correspondence with primary HSCs and that this HSC score is enhanced by the addition of DLL4 and VCAM1 (fig. S8). Using two independent methods including a previously reported “HSC maturation score-card” (*59*) and “Automated Cell Type Identification using Neural Networks” (ACTINN) (*59*, *60*), we determine that our PSC-derived HSCs most strongly resemble HSCs from the Carnegie stage 14/15 AGM (fig. S8).

### Hematopoietic progenitors generated in the presence of DLL4 and VCAM1 are capable of maturation into CD8^+^ T cells

**Fig. 4.**
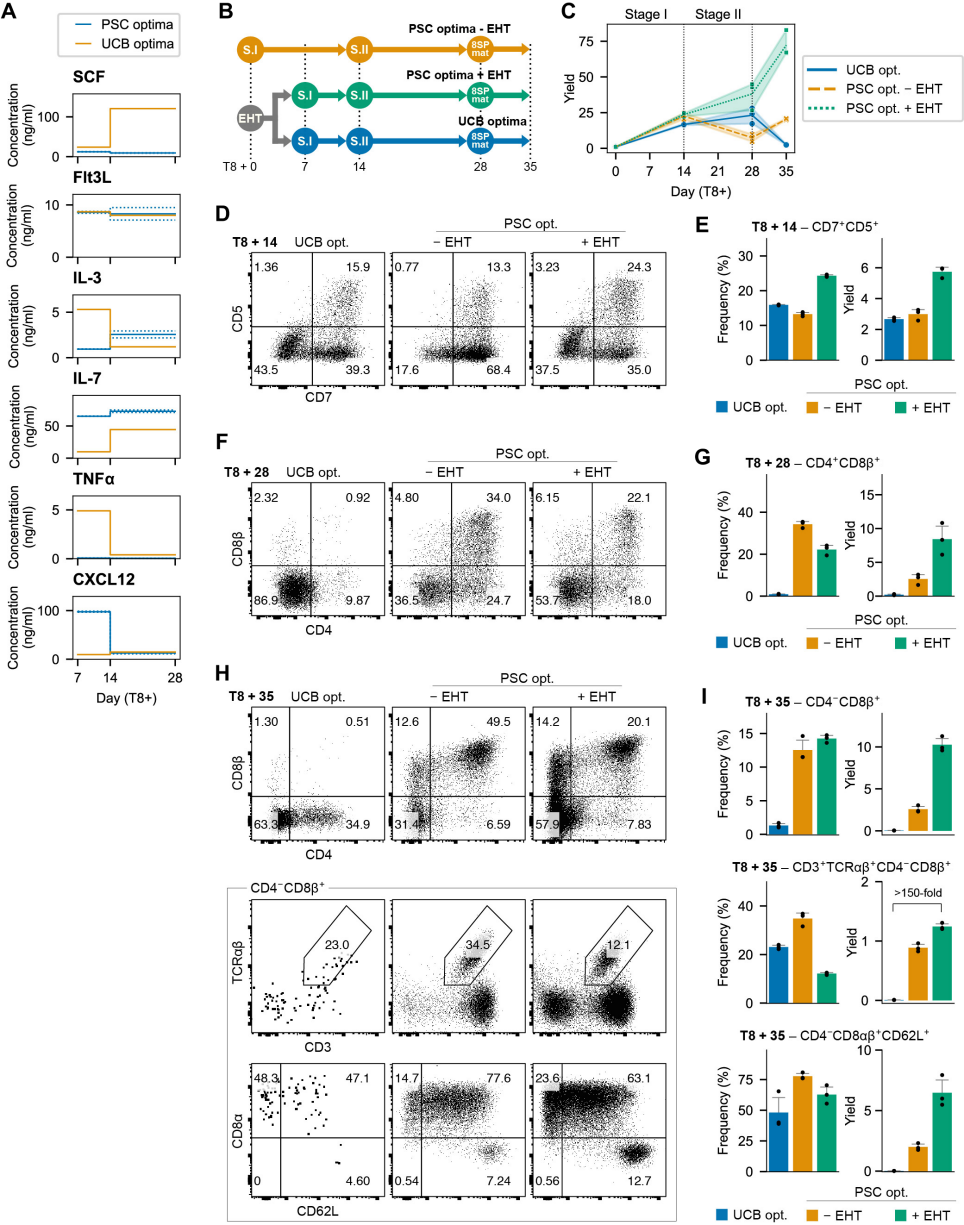
Optimized cytokines enhance T cell development. (**A**) Predicted optima for PSC-derived T cell differentiation compared with previously identified optima for UCB-derived HSPC differentiation to the T lineage. (**B**) To validate predictions, CD34^+^ cells were cultured in EHT conditions for 7 days and then transferred to cytokines optimized for PSC- or cord blood–derived cells. In addition, CD34^+^ cells were placed directly into PSC optima without the EHT step. (**C**) Total viable cell yield for each test condition per input CD34^+^ cell (*n* = 3). (**D**) Representative flow cytometry of CD7^+^, CD5^+^ T cell progenitors at day 8 + 14. (**E**) Quantification of the frequency and yield of CD7^+^, CD5^+^ T cell progenitors (yield is reported as number of CD5^+^, CD7^+^ cells per CD34^+^ input cell, *n* = 3, mean ± SD). (**F**) Representative flow cytometry of CD4^+^, CD8^+^ DP cells at day 8 + 28. (**G**) Quantification of the frequency and yield of CD4^+^, CD8^+^ DP cells (yield is reported as number of CD4^+^, CD8^+^ cells per CD34^+^ input cell, *n* = 3, mean ± SD). (**H**) Representative flow cytometry at day 8 + 35 after stimulation with anti-CD2/3/28 antibodies in the presence of + IL-21. (**I**) Quantification of the frequency and yield of the phenotypes shown in (H). Yield is reported as abundance of indicated cell population per CD34^+^ input cell (*n* = 3, mean ± SD).

After demonstrating that the addition of DLL4 and VCAM1 during EHT is sufficient to promote emergence of CD7^+^, CD5^+^ T cell progenitors, we sought to verify that these cells are able to develop into CD4^+^, CD8^+^ double positive (DP) cells and CD8^+^, CD4^−^, CD3^+^, TCRαβ^+^ mature T cells (Fig. 4, A to G). We transferred PSC-derived hematopoietic progenitors into a serum-free medium supplemented with a cytokine composition that we previously optimized for generating CD4^+^, CD8^+^ DP cells from UCB (Fig. 4B) (*61*). Unfortunately, this medium did not efficiently support T cell maturation from hPSCs. (Fig. 4, F and G). We thus set out to understand how the PSC-derived HSPCs produced in our protocol respond to different cytokine concentrations over time, and use that information to develop an optimized PSC-derived T cell maturation method.

We have previously shown that statistical dose response models are highly effective for optimizing stage-specific differentiation medium formulations (*61*). We conducted a six-cytokine, five-concentration factorial-type experiment over two stages of T cell development (fig. S9). In the first 7 days after EHT, we measured CD5^+^,CD7^+^ proT, CD4^+^,CD8^−^, CD3^−^ immature single positive (CD4ISP), and CD4^+^,CD8^+^,CD3^−^ DP. In the subsequent 14 days, we measured CD3^−^ DP, CD3^+^ DP, and CD4^−^, CD8^+^, CD3^+^ (CD8SP). An equal number of cells were seeded in each test condition, and we measured the absolute number of each cell type at the end of each stage using flow cytometry. We then used this information to derive polynomial dose response models using regression.

Over the 7-day early differentiation stage, proT, CD4ISP and DP, CD3^+^ output had a strong positive response to interleukin-7 (IL-7) concentration and a moderate positive response to CXCL12. Outputs of these populations exhibited a negative response to increasing tumor necrosis factor α (TNFα) concentrations. During the maturation stage, CD3^−^ and CD3^+^ DP as well as CD8SP responded positively to IL-7 and IL-3 and negatively to TNFα (fig. S9). This experimental design also allowed us to examine multiplicative interactions between cytokines (fig. S9).

Next, we sought to determine an optimal combination of cytokine concentrations for each differentiation stage. We defined a desirability function to maximize the number of proT, CD4ISP, and CD3^−^ DP cells for the early stage and CD3^−^, DP, CD3^+^, DP, and CD8SP for the maturation stage (fig. S9). Each desirability function scales the predicted output from our dose response models between [0,1] for a given set of cytokine concentrations. We then combined the value of each desirability function using the geometric mean to calculate an overall desirability score that could be optimized using single-objective optimization algorithms. Each stage was optimized separately using the basin-hopping algorithm (*62*). To avoid settling in local optima, we repeated the procedure with 25 random initializations and retained the top 5 most desirable solutions. The cytokine concentrations that elicited these five responses were then averaged to provide a set of optimal cytokine concentrations for each stage of T cell development from PSC-derived hematopoietic progenitors (Fig. 4A and fig. S9).

We compared these two newly optimized medium formulations (PSC optima) against the previously developed cord blood–optimized medium (UCB optima; Fig. 4, A and B) (*61*). PSC optima improved total cellularity compared to the UCB control as early as day 14, and the magnitude of this effect was amplified over the course of the differentiation (Fig. 4C). Furthermore, the yield of desired cell types was markedly improved by the new medium formulation. The PSC-optimized early-stage medium increased the abundance of CD7^+^, CD5^+^ proT cells twofold compared to the UCB control (Fig. 4, D and E).

Following the maturation stage, PSC optima caused a marked 40-fold improvement in CD4^+^, CD8^+^ DP yields compared to the UCB control (Fig. 4, F and G). We nonspecifically activated TCR signaling with antibodies against CD3, CD28, and CD2 to simulate positive selection and quantified the yield of CD8SP, CD3^+^, TCRαβ^+^ cells. The optimized medium improved the yield by more than 150-fold compared to the UCB optima (Fig. 4, H and I).

Having established optimized and chemically defined T cell maturation conditions specific for PSC-derived cells, we revisited the impact of EHT coating condition on the production of more mature T cell phenotypes after 21 days of T cell differentiation. We demonstrate that the addition of DLL4 during EHT results in a substantial and significant increase in the yield of both CD4^+^,CD8^+^ DP cells (*P* < 0.0001, two-way ANOVA) and CD3^+^, TCRαβ^+^ cells (*P* = 0.0034, two-way ANOVA) compared to uncoated wells or wells coated with VCAM1 alone (fig. S10). At this later time point, we no longer observed a benefit of adding VCAM1 in addition to DLL4 during EHT (fig. S10). This suggests that VCAM1 may act by accelerating the kinetics of pro-T cell development, whereas DLL4 is crucial for increasing the yield of both early T cell progenitors and more mature phenotypes.

We also compared our defined maturation method to the OP9-DL4 stromal system, a conventional platform that has been widely used for T cell differentiation. Notably, by day 21 of T cell differentiation, chemically defined conditions substantially increased the yield of CD4^+^,CD8^+^ DP cells compared to OP9s (27.4-fold; *P* = 0.029, Mann-Whitney test; fig. S10). Furthermore, at day 21 of T cell differentiation, we observed TCRαβ^+^ cells using our defined conditions but did not detect this population using OP9-DL4 at this time point (fig. S10). In the only previously reported stromal- and serum-free T cell differentiation protocol that produced T cells with endogenously rearranged TCRs, TCRαβ^+^ cells were not observed until day 35 of T cell differentiation (*16*). These data show that our defined process leads to faster T cell maturation dynamics compared to previous methods.

**Fig. 5.**
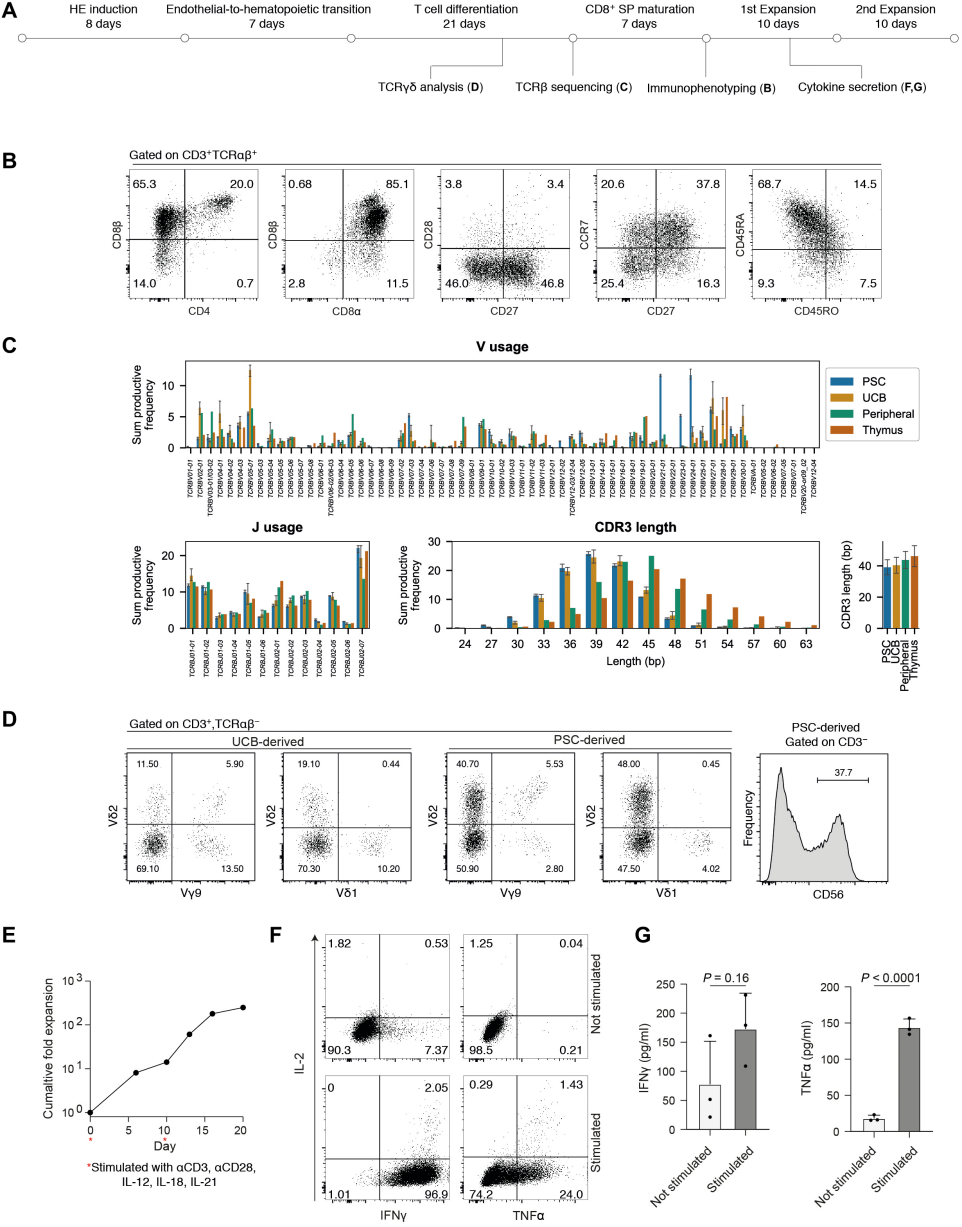
PSC-derived T cells are functional and display a diverse TCR repertoire. (**A**) Schematic overview and timeline of experiments to assess T cell phenotype and function. (**B**) Flow cytometry analysis of mature, PSC-derived T cells followed CD8SP maturation. Plots are representative of *n* = 3 differentiation replicates. (**C**) High-throughput sequencing of TCR β chains from PSC-derived T cell differentiation cultures (*n* = 3 independent differentiation wells). Data for UCB, peripheral blood–derived T cells, and primary thymus were generated and first presented by Edgar *et al.* (*61*) and shown again here for comparison to the PSC-derived cell sequencing, which we report here for the first time. Error bars, ±SD. (**D**) Left: Flow cytometry analysis of Vδ1, Vδ2, and Vγ9 expression of TCRγδ T cells differentiated in vitro from UCB or PSCs. Right: Flow cytometry quantification of CD3^−^, CD56^+^ cells from PSC-derived cells at day 28 of in vitro T cell maturation. Plots are representative of *n* = 3 differentiation replicates. (**E**) Expansion of sorted CD3^+^, TCRαβ^+^ PSC-derived T cells. Two expansion phases were carried out. Each expansion phase begins with 24-hour stimulation with immobilized αCD3 and soluble αCD28 antibodies on RetroNectin-coated plates in the presence of IL-12, IL-15, IL-7, IL-18, and IL-21. After 24 hours, cells were transferred to fresh RetroNectin-coated plates and cultured for 9 days in the presence of IL-15 and IL-7, with the remaining cytokines and αCD3 αCD28 antibodies removed. (**F**) Following 7 days of expansion, cells were assayed for production of effector proteins in the presence or absence of nonspecific PMA/ionomycin stimulation. Intracellular flow cytometry is representative of three stimulation replicates. (**G**) Enzyme-linked immunosorbent assay (ELISA) analysis of the growth medium from cells stimulated with PMA/ionomycin and unstimulated controls. Mean ± SD, *n* = 3 replicates, *P* values from unpaired *t* tests.

We comprehensively characterized the immune phenotype, TCR repertoire, and functional properties of the mature T cells generated using our defined and optimized protocol. One limitation of previous in vitro differentiation methods is that they tend to produce T cells with unconventional immunophenotypes. A common unintended product is cells that express a CD8αα homodimer, a characteristic feature of innate-like cells (*10*, *14*). Furthermore, PSC-derived TCRαβ^+^ T cells have previously been reported to lack robust expression of the adhesion molecule CD62L and the chemokine receptor CCR7 (*10*, *14*). The TCRαβ^+^ T cells generated in our protocol expressed the conventional CD8αβ heterodimer and robustly expressed CD62L (Fig. 4H) as well as CCR7 (Fig. 5, A and B). These cells were predominantly positive for CD45RA, a marker of a naïve phenotype (Fig. 5B).

The PSC-derived T cells produced by our method expressed a diverse TCR repertoire with broad TCRV and TCRJ usage (Fig. 5C). While the TCR diversity of PSC-derived T cells was generally comparable to T cells differentiated from UCB and primary thymocytes, we observed an increased usage of *TCRBV21-01*, *TCRBV23-01*, and *TCRBV24-01* (Fig. 5C). A previous analysis of *TCRBV* usage over developmental time showed enrichment for *TCRBV24-01* in CD4^−^, CD8^−^ DN progenitors (*63*). The observed prevalence of these *TCRBV* segments in our data is consistent with the fact that the PSC-derived progenitors that we sequenced contain a higher proportion of DN cells compared to what would be found in the thymus or in the UCB samples we sequenced previously. The mean CDR3 length for PSC-derived cells was slightly shorter than postnatal thymus and closer to the median CDR3 length in T cells differentiated from cord blood progenitors (Fig. 5C). This profile is consistent with a fetal, rather than postnatal, identity.

In addition to TCRαβ^+^ cells, our protocol also generated TCRγδ cells and a small population of CD3^−^, CD56^+^ cells, confirming that multiple lymphoid cell types can be produced in our cultures (Fig. 5D). A recent report established differentiation conditions that copy important aspects of murine yolk sac hematopoiesis using hPSCs (*32*). These yolk sac–like progenitors were able to differentiate into TCRγδ cells that express Vδ2^+^ but not Vδ1^+^ (*32*). In our differentiation system, we observe both Vδ1^+^ and Vδ2^+^, although the ratio of Vδ1^+^ to Vδ2^+^ expressing cells was lower in PSC-derived cells compared to cord blood–derived T cells differentiated in vitro (Fig. 5D).

To assay functionality, we sorted TCRαβ^+^, CD3^+^ cells generated by our differentiation platform and subjected them to αCD3, αCD28 stimulation in the presence of cytokines documented to support expansion of PSC-derived T cells (*13*). We observed ~250-fold cumulative expansion over two successive 10-day expansion phases (Fig. 5E). Intracellular flow cytometry revealed that a large proportion of these T cells produced TNFα and IFNγ and a small proportion produced IL-2 in response to nonspecific stimulation with phorbol 12-myristate 13-acetate (PMA)/ionomycin (Fig. 5F). This expression profile is consistent with a TC1 T cell phenotype, a subtype that is generated in the presence of IL-12 (*64*). We verified functional cytokine secretion by enzyme-linked immunosorbent assay (ELISA) (Fig. 5G). Collectively, these data demonstrate that PSC-derived hematopoietic progenitors that were generated in the presence of DLL4 and VCAM1 were capable of efficient differentiation not only into T cell progenitors but also into mature and functional CD8SP, CD3^+^, TCRαβ^+^ cells.

The engineered EHT niche and optimized T cell differentiation medium that we establish here each substantially improve PSC-derived T cell production. Together, these improvements constitute a highly efficient platform that will enable both fundamental studies of human T lymphopoiesis and scalable T cell manufacturing protocols for cell therapy applications.

## DISCUSSION

In this study, we present a substantially improved serum- and feeder-free method for generating hematopoietic progenitors and T cells from hPSCs. Two other feeder-free hPSC–to–T cell differentiation systems have been reported to date (*13*, *16*). The method presented by Iriguchi *et al.* (*13*) does not support endogenous αβTCR recombination, which is an important limitation that precludes research into key stages of T cell development. Our approach generates functional αβT cells with a diverse TCR repertoire.

The protocol presented by Trotman-Grant *et al.* (*16*) supports αβTCR recombination but lacks an explicit EHT culture phase. Here, we find that the addition of an EHT stage comprising DLL4 and VCAM1 markedly improves progenitor T cell differentiation by increasing the frequency of T lymphoid–competent CD34^+^ cells by an order of magnitude. Our EHT culture phase provides the minimal necessary signals required for emergence of HSPC with T cell potential (*22*, *65*). Because the system is both simple and chemically defined, it allowed us to dissect the contributions of individual molecules during EHT, and to determine that, in addition to supporting T cell potency, DLL4 decreases neutrophil and megakaryocyte output and increases the frequency of erythroid progenitors. Using this EHT platform, we also learned that VCAM1 synergizes with DLL4 to accelerate the emergence of T lymphoid progenitors. Control over the niche composition and the resulting cellular output that is afforded by our system will aid future cell manufacturing efforts. In addition, the differentiation platform we have developed will enable future studies into the intermediate cell states that bridge pluripotent stem cells to T cells.

Here, we verify previous reports that notch signaling during EHT is critical for emergence of HSC/MPP with T lineage potential (*24*, *27*). While DLL4 might also affect HSC/MPP once they have already emerged, notch inhibition experiments demonstrate that this pathway is indispensable during the EHT process itself (*27*). Surface phenotyping indicated an increase in the frequency of phenotypic HSCs in the uncoated EHT condition. In contrast, scRNA-seq demonstrates that DLL4 increases the proportion of cells that can be transcriptionally classified as HSCs. Using our scRNA-seq data, we observed an increase in HSC frequency upon the addition of DLL4 either using our own cell type annotations or by globally integrating our data with two previously published and annotated datasets from the primary FL (*58*) and AGM (*59*). While the flow cytometry–based phenotyping strategy used in our study has previously been used to enrich for true definitive HSCs from primary cell sources, such as UCB, this marker panel has not been functionally validated for use with PSC-derived hematopoietic cells (*34*). Given that the results of in vitro erythroid/myeloid differentiation, CFU-GEMM quantification, and T cell differentiation experiments track very strongly with our transcriptome-based cell type quantifications, we believe that scRNA-seq is a more useful cell type classification strategy than surface marker flow cytometry in this context.

We show that VCAM1 cooperates with DLL4 to enhance the magnitude of notch signaling during the EHT culture phase. Unexpectedly, we also found that VCAM1 promotes inflammatory-responsive gene expression in nascent HSC/MPP. Studies in zebrafish and mouse have shown that inflammatory signals are important for HSC emergence, both upstream and downstream of the notch pathway (*66*–*69*). The differentiation method we present here will enable future interrogation into the role of inflammatory signaling during human EHT.

When we first attempted to differentiate hPSC-derived T cell progenitors into mature T cells using a medium that we previously optimized for cord blood–derived progenitors, we were largely unsuccessful. Upon reoptimizing the medium for hPSC-derived cells, we observed >150-fold increase in mature T cell production. Notably, the PSC-optimized medium uses the exact same cytokines as the cord blood medium and these formulations differ only in their concentrations. These distinct optima may reflect differences in the developmental stage and transcriptional state of the starting material. PSC-derived cells required higher concentrations of CXCL12, which may be necessary to produce and maintain early lymphoid progenitors that are already abundant in cord blood (*70*). PSC-derived cells also required markedly less of the inflammatory cytokine TNFα compared to cord blood. The inflammatory program promoted by VCAM1 during EHT may lessen the subsequent requirement for inflammatory cytokines in the medium.

The early T cell differentiation medium that we optimized for PSCs can partially replace the EHT medium, albeit at markedly reduced efficiency. Future work will determine whether the early T cell medium can actually promote EHT of HE cells, or whether it simply allows the very small number of hematopoietic cells present in the day 8 aggregates to efficiently differentiate toward the T lineage.

Overall, the optimized differentiation medium we have established allows us to produce ~1 CD8SP, CD3^+^, TCRαβ^+^ cell per PSC-derived CD34^+^ cell before expansion. This remains less efficient than using cord blood cells as starting material, which produce approximately 40 CD8SP, CD3^+^, TCRαβ^+^ cells per input CD34^+^ cell (*61*). The addition of an expansion phase allowed us to generate ~300 T cells per input PSC-derived CD34^+^ cell. A therapeutic dose of CAR T cells is on the order of 2 × 10^8^ cells (*71*). Such a dose could theoretically be achieved with ~700,000 PSC-derived CD34^+^ cells, a number that is readily attainable from a six-well plate of aggregates. Subsequent differentiation to the preexpansion CD8SP stage could then be carried out in a T-175 flask, 15-cm^2^ dish or equivalent. Future process optimization and scale-up will enable translation of this protocol to the clinic.

The method we report here has widespread applicability beyond manufacturing T cell therapies. We have shown that a defined EHT process allows us to study the impact of juxtracrine factors on the transcriptome and lineage potential of emergent HSPCs. In another application, we used this method to study how cytokine dosages affect T cell maturation. Although we developed this platform to achieve robust, efficient, and customizable T cell differentiation from hPSC, we have shown that this method can be used to produce and study multiple hematopoietic cell types. In addition to producing T cell progenitors, the defined EHT process also generates erythroid/megakaryocyte and myeloid progenitors and will enable basic research into blood stem cell emergence and lineage specification.

## METHODS

### hPSC culture

The human iPSC cell line iPS11 (Alstem Cell Advancements, episomally derived from human foreskin fibroblasts) was cultured on tissue culture–treated plasticware precoated with 1 ml per well Geltrex (Life Technologies, A1413302) for 1 hour at 37°C. Cells were cultured in serum-free medium (mTeSR1, STEMCELL Technologies, 85850) that was supplemented with penicillin-streptomycin (0.5% v/v, Invitrogen, 15140122). The growth medium was aspirated and replenished daily, and cells were maintained at 37°C, 5% CO_2_. Following thawing or passaging, the medium was additionally supplemented with the ROCK inhibitor Y-27632 (STEMCELL Technologies, 72308) at 7.5 and 5 μM concentrations, respectively. To passage, cells were partially dissociated to small aggregates with 1× TrypLE Express (Thermo Fisher Scientific, 12605028) at 37°C followed by cell scraping. Dissociation was carried out for 2 to 4 min.

### Aggregation and CD34^+^ induction

For CD34^+^ induction medium formulations, see workbook S1. iPS11 cells were grown to ~90% confluency and dissociated to single cells with TrypLE Express for 3 to 5 min at 37°C. Dissociated cells were collected and counted, and a desired cell number was pelleted at 200*g* for 5 min. Following supernatant aspiration, cells were resuspended in 2 ml of T0 medium per well and deposited into AggreWell 400 six-well plates (STEMCELL Technologies, 34425) prepared with AggreWell Rinsing Solution (STEMCELL Technologies, 07010) according to the manufacturer’s instructions. Cells were seeded at a density of 1.26 × 10^6^ cells per well of a six-well plate and aggregated by centrifugation at 200*g* for 5 min. For the duration of the CD34^+^ induction, cells were cultured at 37°C in a hypoxia incubator at 5% CO_2_, 5% O_2_.

Twenty-four hours after seeding, 2 ml of the T1 medium was added to each well. Forty-two hours after initiating the differentiation, the medium was aspirated and replaced with the T1.75 medium (2 ml per well). The following day, an additional 2 ml of the T1.75 medium was added to each well. Ninety-six hours after initiating the differentiation, the T1.75 medium was aspirated and replaced with the T4 medium (2 ml per well). Forty-eight hours later, the medium in each well was supplemented with 2 ml of T6 medium. The following day, a subset of aggregates was harvested and dissociated with TrypLE Express, washed with 200 μl of HF buffer [Hanks’ balanced salt solution (HBSS), Thermo Fisher Scientific, 14175103, supplemented with 2% fetal bovine serum (FBS), Thermo Fisher Scientific, 12483020], stained with antibodies against CD34, CD43, CD73, and CD184, and analyzed by flow cytometry.

One hundred ninety-two hours after initiating the differentiations, aggregates were collected and pelleted by centrifugation at 200*g* for 5 min. The spent medium was aspirated, and aggregates were dissociated in 3 ml of TrypLE supplemented with deoxyribonuclease I (MilliporeSigma, 260913-10MU) for 10 to 15 min. Cells were pipetted to a single-cell suspension, washed, and counted. CD34^+^ cells were enriched using the CD34-positive selection kit (Miltenyi Biotec, 130-046-702) according to the manufacturer’s instructions. CD34^+^-enriched cells were cryopreserved using CryoStor CS10 (STEMCELL Technologies, 07930) for use in downstream culture.

### EHT culture

CD34^+^ cells generated above were used as input for EHT culture. Coating solution was prepared using sterile phosphate-buffered saline (PBS) combined with Fc-tagged DLL4 (15 μg/ml; Sino Biological, 10171-H02H) and Fc-tagged VCAM1 (2.5 μg/ml; R&D Systems, 643-VM). Tissue culture–treated 96-well plates (Fisher Scientific, 12-556-008) were precoated with 50 μl of coating solution overnight at 4°C. Coating solution was aspirated, and plates were washed with PBS immediately before use. CD34^+^-enriched cells were resuspended in EHT medium (workbook S1) at a concentration of 1 × 10^5^ cells/ml unless otherwise indicated. Cells (10,000/100 μl) were seeded onto each well of the 96-well plate unless otherwise indicated. Cultures were incubated at 37°C, 5% CO_2_ for 5 or 7 days as specified in the main text, and nonadherent cells were harvested by gentle pipetting.

### T cell differentiation

Nonadherent cells harvested from EHT cultures were pelleted by centrifugation at 300*g* for 5 min. For the data shown in Fig. 1, the spent medium was aspirated and cells were resuspended in an unoptimized T cell differentiation medium at a split ratio of 1:3. The unoptimized T cell differentiation medium was prepared by combining Iscove’s modified Dulbecco’s medium (IMDM) with GlutaMAX basal medium (Thermo Fisher Scientific, 31980030) with 20% BIT 9500 serum substitute (STEMCELL Technologies, 09500), 0.05% low-density lipoprotein (STEMCELL Technologies, 02698), 60 μM ascorbic acid (Sigma-Aldrich, A8960), 24 μM 2-mercaptoethanol (Sigma-Aldrich, M3148), stem cell factor (SCF) (0.02 μg/ml; R&D Systems, 255-SC), Flt3L (0.02 μg/ml; R&D Systems, 308-FK), TPO (Thrombopoietin) (0.02 μg/ml; R&D Systems, 288-TP), IL-7 (0.02 μg/ml; R&D Systems, 207-IL), IL-3 (0.01 μg/ml; R&D Systems, 203-IL), and TNFα (0.005 μg/ml; R&D Systems, 210-TA). Cells were seeded in 100 μl per well of a 96-well plate precoated with DLL4 and VCAM1, as described above. Three to 4 days after seeding, cells were fed with an additional 100 μl per well of unoptimized T cell differentiation medium. Seven days after seeding, cells were harvested by pipetting and seeded onto freshly coated plates in unoptimized T cell differentiation medium and cultured for an additional 7 days, with a medium top-up after 3 days.

For the experiments described in Fig. 4, cells were cultured as described above but using either cord blood–optimized early and late T cell differentiation medium or PSC-optimized early and late T cell differentiation medium (workbook S1). After EHT, cells were split 1:3 and seeded into 96-well plates precoated with DLL4 and VCAM1 in 100 μl of early T cell differentiation medium per well for 7 days with a 100-μl top-up on day 3 or 4. After 7 days, cells were split 1:3 into 96-well plates freshly coated with DLL4 and VCAM1 as described above in late T cell differentiation medium where they were cultured for 14 days with a 100-μltop-up on day 3 or 4 followed by half-medium changes twice per week. All T cell differentiation cultures were carried out at 37°C, 5% CO_2_.

After 7 days of EHT culture and 21 days of T cell differentiation, T cells were stimulated to promote DP to CD8SP maturation. Stimulation was carried out in 96-well plates precoated with DLL4 and VCAM1 as described above in late T cell differentiation medium (100 μl per well) supplemented with IL-21 (20 ng/ml; R&D Systems, 8879-IL) and 1.25% ImmunoCult Human CD3/CD28/CD2 T cell activator (STEMCELL Technologies, 10970). After 48 hours, each well was topped up with an additional 100 μl of late T cell differentiation medium with IL-21 but without the T cell activator reagent where they were cultured for an additional 5 days.

### Extended myeloid and erythroid differentiation

Nonadherent cells harvested from day 5 EHT cultures were pelleted by centrifugation at 300*g* for 5 min. Cells were counted using a hemocytometer and seeded at 2500 cells per well of a 96-well plate in 200 μl of erythroid/myeloid supporting medium (IMDM supplemented with GlutaMAX, Thermo Fisher Scientific, 31980030), 30% FBS, 1% bovine serum albumin (Wisent Bioproducts, 809-098-EL), 0.1 mM 2-mercaptoethanol (Sigma-Aldrich, M3148), IL-6 (20 ng/ml; R&D Systems, 206-IL), SCF (50 ng/ml; R&D Systems, 255-SC), IL-3 (20 ng/ml; R&D Systems, 203-IL), G-CSF (20 ng/ml; R&D Systems, 214-CS), GM-CSF (20 ng/ml; R&D Systems, 215-GM), and EPO (Erythropoietin) (3 U/ml; R&D Systems, 287-TC). Cells were cultured for 7 days followed by EHT and assessed by flow cytometry.

### Colony-forming assays

Nonadherent cells harvested from day 5 EHT cultures were pelleted by centrifugation at 300*g* for 5 min. Cells were counted using a hemocytometer and seeded in 35-mm dishes at 500 cells per dish in MethoCult H4435 Enriched (STEMCELL Technologies, 04435) according to the manufacturer’s instructions. CFU-GM, CFU-E, and CFU-GEMM colony identification was performed 14 days after seeding in MethoCult medium according to the Atlas of Human Hematopoietic Colonies (STEMCELL Technologies, 28700).

### Flow cytometry

Samples were collected in 96-well V bottom plates and pelleted at 300*g* for 5 min. Supernatant was aspirated, and cells were washed in 200 μl of PBS per well (Thermo Fisher Scientific, 10010049). Cells were incubated for 15 min at room temperature in the dark in 50 μl of PBS with Fc Block (1:100 dilution, BD, 564220) and Zombie ultraviolet dye (1:500 dilution, BioLegend, 423108). Next, 50 μl of antibody cocktail made up at 2× working concentration in HF buffer (HBSS, Thermo Fisher Scientific, 14175103, supplemented with 2% FBS, Thermo Fisher Scientific, 12483020) was mixed 1:1 with the cell suspension. Cells were stained for 30 min on ice protected from light and washed twice with 200 μl of HF buffer per well. Samples were analyzed on a CytoFLEX LX cytometer (Beckman), compensation was performed in CytExpert v2.3, and data were gated and plotted in FlowJo v9. Antibodies used are listed in workbook S1.

### Sample preparation for scRNA-seq

EHT cultures were initiated and carried out as described above. Three wells of a 96-well flat-bottom tissue culture-treated plate were prepared for each coating condition, and cells were cultured in EHT medium for 5 days. On day 5, nonadherent cells were harvested by gentle pipetting. Well replicates were pooled for each condition and pelleted by centrifugation at 300*g* for 5 min. Samples were washed with 200 μl of HF buffer (HBSS, Thermo Fisher Scientific, 14175103, supplemented with 2% FBS, Thermo Fisher Scientific, 12483020). Cells were stained with 0.1 μg of TotalSeq-B anti-human Hashtag antibody (BioLegend, 394633, 394635, 394637, or 394639) per sample diluted in 100 μl of HF buffer for 30 min on ice. Cells were washed three times with HF buffer. Cell density was determined by hand counting using a hemocytometer, and samples were pooled at equal cell ratios per sample. The pooled sample was counted and loaded on the chromium controller as per the manufacturer’s instructions aiming for a target cell capture of 5900 cells.

### scRNA-seq analysis

The Cell Ranger (*35*) function mkfastq was used for FASTQ generation, and Cell Ranger count was applied using default parameters for trimming, alignment, and counting using hg19 as the reference genome. The data were further processed in python using the scanpy package (*36*) (version 1.7.0). Demultiplexed sample antibody counts from the Cell Ranger pipeline were used to identify and filter out empty droplets and doublets. Doublets were then further identified and filtered by removing cells that had greater than 36,000 counts or that had greater than 6000 genes. Dying/dead cells were removed by filtering cells that had >18% mitochondrial reads. This filtering step removed 159 cells (2% of the total). Genes that were expressed in few cells (*n* = 3) were also removed from the processed dataset. The fully filtered dataset contained 7589 cells and 20,546 genes with a median of 4190.36 genes per cell. The expression matrix was then normalized to 1 × 10^4^ counts per cell and was log-transformed. A total of 1672 highly variable genes were detected and used to perform principal components analysis. Forty principal components were used to generate a neighbors graph. Unsupervised Leiden clustering was performed, and a Uniform Manifold Approximation and Projection (UMAP) embedding was generated for dataset visualization.

Cell cycle signatures in the dataset were annotated using a previously reported gene list (*72*), and the G_2__M and G_1__S signatures were regressed using the scanpy regress_out function to remove cell cycle contributions to the expression matrix. Leiden clusters (resolution = 0.18) were annotated using marker genes as indicated in the main text. Differential expression was performed with the scanpy package using a Wilcoxon rank sum test (*36*). Differential expression was performed between cell types and between plating conditions within the MPP cells. Reported *P*_adjusted_ values were calculated using Benjamini-Hochberg correction.

Regulons were computed with the pySCENIC pipeline (*44*). Grnboost2 was used to determine coexpression modules, and hg19 transcription factor motifs provided by the Aerts laboratory were used to identify direct transcription factor motif interactions. Regulon activity was quantified in single cells using the area under curve metric built into the pipeline. Differential regulon activity was computed using the SCENIC regulon specificity score and with scanpy’s rank_genes_groups function (*36*, *73*).

Pathway analysis was performed using the gseapy package’s (v0.10.5) single-sample gene set enrichment analysis (ssgsea) function and the Biocarta pathway database provided by the gseapy package (*74*, *75*). Reported *P*_adjusted_ values were calculated using Benjamini-Hochberg correction.

### scRNA-seq integration with primary datasets

To determine probable cell types, we used a transfer-based approach implemented in the R package “Seurat” (*57*). For our dataset and a reference dataset from primary human FL (*58*), we first identified variable features using the “FindVariableFeatures” function with default settings, followed by principal components analysis. Transfer anchors were then identified between each reference and our data using the “FindTransferAnchors” function based on the top 30 principal components in each. The “TransferData” function was then used to compute prediction scores for each class on our dataset. We classified PSC-derived cells into the FL cell type for which it scored the highest. We repeated this procedure for the dataset from Calvanese *et al.* (*59*). In addition, we integrated PSC-derived cells with UMAPs comprising cells from Calvanese *et al.* (*59*) using the Scanpy “Ingest” function with default settings. We applied ACTINN (*60*) using the same training dataset and input parameters as previously reported by Calvanese *et al.* (*59*) to classify our PSC-derived HSCs in fig. S8H.

### Cytokine dose response models during T cell development

Cytokine dose responses were measured using a six-factor orthogonal central composite design (CCD) experiment using JMP14 software. The CCD combines a two-level fractional factorial, where concentrations are scaled to (−1,1), with added center (0) and axial (−α,α) points. The value of α = 2.366 was chosen automatically by JMP14 to ensure orthogonality. This resulted in a five-level design capable of estimating all linear, quadratic, and two-factor interaction terms. Select third-order terms were also included where they improved model fit. Cytokine concentrations were scaled logarithmically to provide a continuous mapping between actual and scaled values. Experiments were laid out using an epMotion 5073 liquid handler (Eppendorf). For the first stage, cells were passaged after EHT to test conditions at equal densities and cultured for 7 days. For the second stage, cells after EHT were cultured with cytokine concentrations able to support T lineage induction and cultured for 7 days. Subsequently, these cells were pooled and passaged to test conditions and cultured for an additional 14 days. At the end of each experiment, phenotype and absolute cell numbers were measured using flow cytometry. The number of cells in each population of interest was transformed as $\sqrt{X}$ or log(*X* + 1), and dose response models were fit using least-squares regression. The concentrations of cytokines tested are provided in table S1. Calculated regression coefficients are provided in tables S2 to S7.

### Optimizing dose response models for generating mature T cells

Regression coefficient estimates were used to construct polynomial models using custom code written in Python 3.7. Desirability functions were applied to each model to maximize the response of that cell population, and an overall desirability was calculated at the geometric mean of each individual desirability score (*76*). The overall desirability was then optimized using the basin-hopping algorithm (*62*) from the SciPy Python library. The solution space was constrained to be within a hypersphere with radius α = 2.366 to ensure that optimal solutions were not extrapolations beyond the range of cytokine concentrations tested. Within this space, the optimizer was randomly initialized 25 times, and the top five most desirable solutions were averaged to provide an optimal set of cytokine concentrations (workbook S1). The code used for optimization was previously reported by Edgar *et al.* (*61*) and is available at gitlab.com/stemcellbioengineering/polynomialfeatures (v1.1).

### TCR repertoire sequencing

For TCR sequencing, PSC-derived CD34^+^ cells were subjected to EHT in the presence of DLL4 and VCAM1 as described above. Nonadherent hematopoietic cells were differentiated into T cells using the optimized conditions described above. After 21 days of T cell differentiation, 1 × 10^6^ cells per sample were pelleted at 400*g* for 6 min and washed once with PBS. Genomic DNA was extracted using QuickExtract DNA Extraction Solution (Lucigen, QE9050) following the manufacturer’s instructions and diluted in tris-EDTA. TRB ImmunoSEQ survey resolution sequencing was performed by Adaptive Biotechnologies. TRB sequences from PSC-derived cells were compared to reference data previously acquired and reported by Edgar *et al.* (*61*).

### OP9-DL4 coculture

OP9-DL4 stromal cells were a gift from C. Sturgeon. OP9-DL4 cells were maintained and passaged in minimum essential medium α (MEMα) (Thermo Fisher Scientific, 12561056) supplemented with 15% HyClone FBS (Thermo Fisher Scientific, SH3039603) and 1% penicillin-streptomycin. For T cell differentiation cocultures, 10,000 PSC-derived HSPCs were seeded onto a confluent layer of OP9-DLL4 per well of a 24-well plate in MEMα with 20% HyClone FBS, 1% penicillin-streptomycin, IL-7 (5 ng/ml; R&D Systems, 207-IL), and Flt3L (5 ng/ml; R&D Systems, 308-FK). For the first 7 days of coculture only, the medium was additionally supplemented with SCF (30 ng/ml; R&D Systems, 255-SC). Cocultures were passaged over 4 or 5 days. To passage, cells were mechanically dissociated by pipetting and passed through a 40-μm cell strainer. Strained cells were pelleted for 5 min at 500*g* and resuspended in freshly prepared coculture medium before seeding onto OP9-DL4 cells seeded the previous day.

### T cell expansion

Following defined T cell differentiation in optimized conditions and CD8SP maturation as described above, CD8β^+^, CD4^−^, CD3^+^, TCRαβ^+^ were sorted using the MoFlo Astrios Cell Sorter (Beckman Coulter). The day before sorting, a plate coating solution was prepared using sterile PBS combined with anti-CD3 antibody (3 μg/ml; BioLegend, 317326) and RetroNectin (150 μg/ml; Takara Bio Inc., T100B). Tissue culture–treated 96-well plates (Fisher Scientific, 12-556-008) were precoated with 50 μl of coating solution overnight at 4°C. Coating solution was aspirated, and plates were washed with PBS immediately before use. The complete expansion medium was prepared by combining IMDM with GlutaMAX basal medium (Thermo Fisher Scientific, 31980030) with 4% B-27 supplement minus vitamin A (Thermo Fisher Scientific, 12587010), 0.50% (v/v) penicillin-streptomycin (Invitrogen, 15140122), 60 μM ascorbic acid (Sigma-Aldrich, A8960), 24 μM 2-mercaptoethanol, IL-7 (0.005 μg/ml; R&D Systems, 207-IL), IL-15 (0.005 μg/ml; R&D Systems, 247-ILB), IL-12 (0.5 μg/ml; R&D Systems, 219-IL), IL-18 (0.5 μg/ml; R&D Systems, 9124-IL), IL-21 (0.2 μg/ml; R&D Systems, 8879-IL), 100 μM Pan Caspase Inhibitor Z-VAD-FMK (R&D Systems, FMK001), and anti-CD28 antibody (3 μg/ml; BioLegend, 302934). Sorted cells were counted using a hemocytometer and seeded onto precoated plates at 75,000 cells per well in 100-μl expansion medium. Twenty-four hours after seeding, cells were harvested, pelleted by centrifugation for 300*g* for 5 min, and resuspended in fresh IMDM supplemented with GlutaMAX basal medium (Thermo Fisher Scientific, 31980030) with 4% B-27 supplement minus vitamin A (Thermo Fisher Scientific, 12587010), 0.50% (v/v) penicillin-streptomycin (Invitrogen, 15140122), 60 μM ascorbic acid (Sigma-Aldrich, A8960), 24 μM 2-mercaptoethanol, IL-7 (0.005 μg/ml; R&D Systems, 207-IL), and IL-15 (0.005 μg/ml; R&D Systems, 247-ILB). Cells were then reseeded onto plates coated with RetroNectin only. Cells were passaged on days 3 and 6. After 10 days, cells were restimulated following the same protocol for an additional 10 days.

### Intracellular flow cytometry and ELISA

To assess IL-2, TNFα, and IFNγ production, we harvested T cells 7 days after the initiation of expansion and subjected them to nonspecific stimulation. A total of 100,000 cells were seeded in a round-bottom 96-well plate in IMDM with GlutaMAX basal medium (Thermo Fisher Scientific, 31980030) with 4% B-27 supplement minus vitamin A (Thermo Fisher Scientific, 12587010), 0.50% (v/v) penicillin-streptomycin (Invitrogen, 15140122), 60 μM ascorbic acid (Sigma-Aldrich, A8960), 24 μM 2-mercaptoethanol with or without PMA (25 ng/ml; Sigma-Aldrich, P8139), and ionomycin (1 μg/ml; Sigma-Aldrich, I0634). For intracellular flow cytometry, brefeldin A (3 μg/ml; eBioscience, 00-4506-51) was added to each well after 1 hour. Cells designated for flow cytometry were harvested 6 hours after stimulation. For ELISA, the medium was harvested from cells 24 hours after stimulation. IFNγ was measured using the IFNγ Human ELISA Kit (Thermo Fisher Scientific, KHC4021), and TNFα was measured using the Novex TNF Human ELISA Kit (Thermo Fisher Scientific, KHC3011), both according to the manufacturer’s instructions.

## Supplementary Materials

### Other Supplementary Material for this manuscript includes the following:

Supplementary Workbook 1

## Notes

View/request a protocol for this paper from *Bio-protocol*.
