# Supplementary material for: DLL4 and VCAM1 enhance the emergence of T cell–competent hematopoietic progenitors from human pluripotent stem cells
Source: Sci Adv. 2022 Aug 24;8(34):eabn5522. doi: 10.1126/sciadv.abn5522 (PMC9401626; doi:10.1126/sciadv.abn5522)
Supplement: Supplementary file 1 — Figs. S1 to S10 Tables S1 to S7 [file sciadv.abn5522_sm.pdf]

Science Advances
AAAS

Supplementary Materials for

DLL4 and VCAM1 enhance the emergence of T cell–competent hematopoietic progenitors from human pluripotent stem cells

Yale S. Michaels *et al.*

Corresponding author: Peter W. Zandstra, peter.zandstra@ubc.ca

Sci. Adv. **8**, eabn5522 (2022)
DOI: 10.1126/sciadv.abn5522

The PDF file includes:

Figs. S1 to S10
Tables S1 to S7
Legend for Supplementary Workbook 1

Other Supplementary Material for this manuscript includes the following:

Supplementary Workbook 1

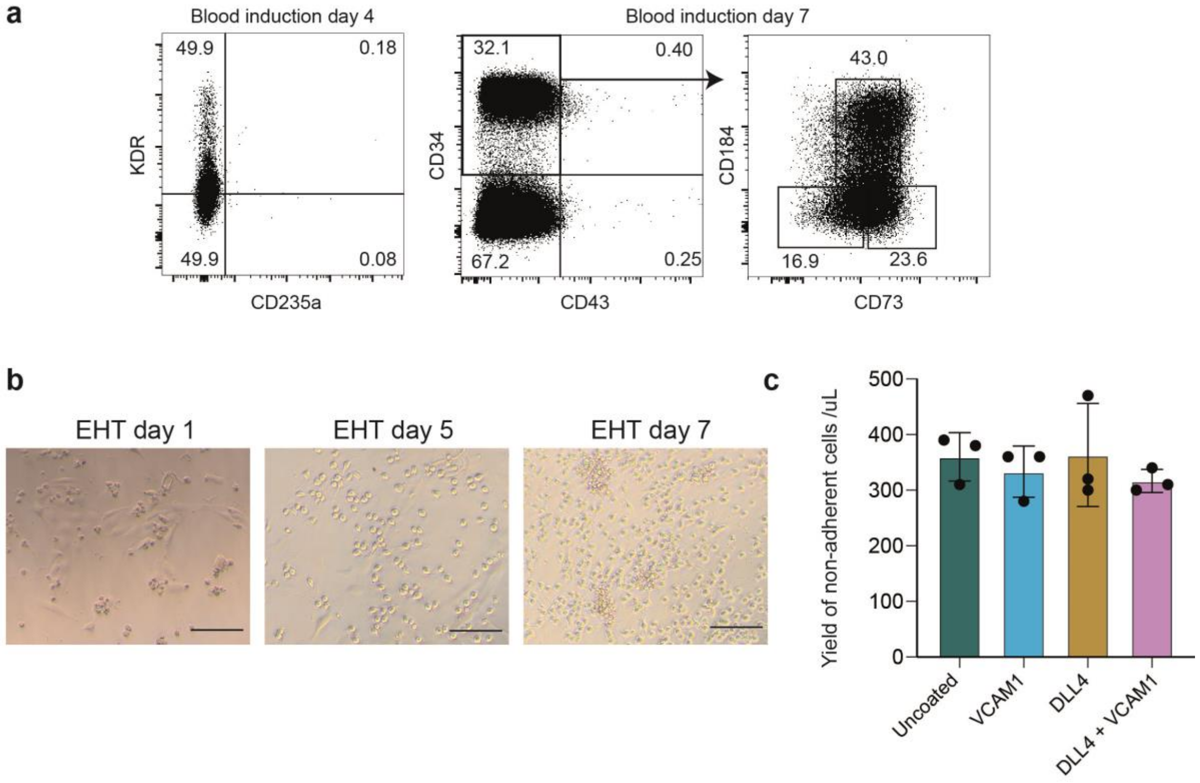

Supplementary Figure 1: Hemogenic endothelium production and endothelial to hematopoietic transition from PSCs. a.) Flow cytometry analysis of mesoderm and hemogenic endothelium generated from PSCs. The absence of the yolk-sac marker CD235a is indicative of a definitive developmental program. **b.)** Bright field images of PSC derived CD34+ cells seeded into EHT cultures in the presence of immobilised DLL4 and VCAM1 at the timepoints indicated. Scale bars = 100um **c.)** The yield of non-adherent hematopoietic cells was quantified after 5 days in EHT under the coating conditions indicated (n=3, mean +/- s.d.).

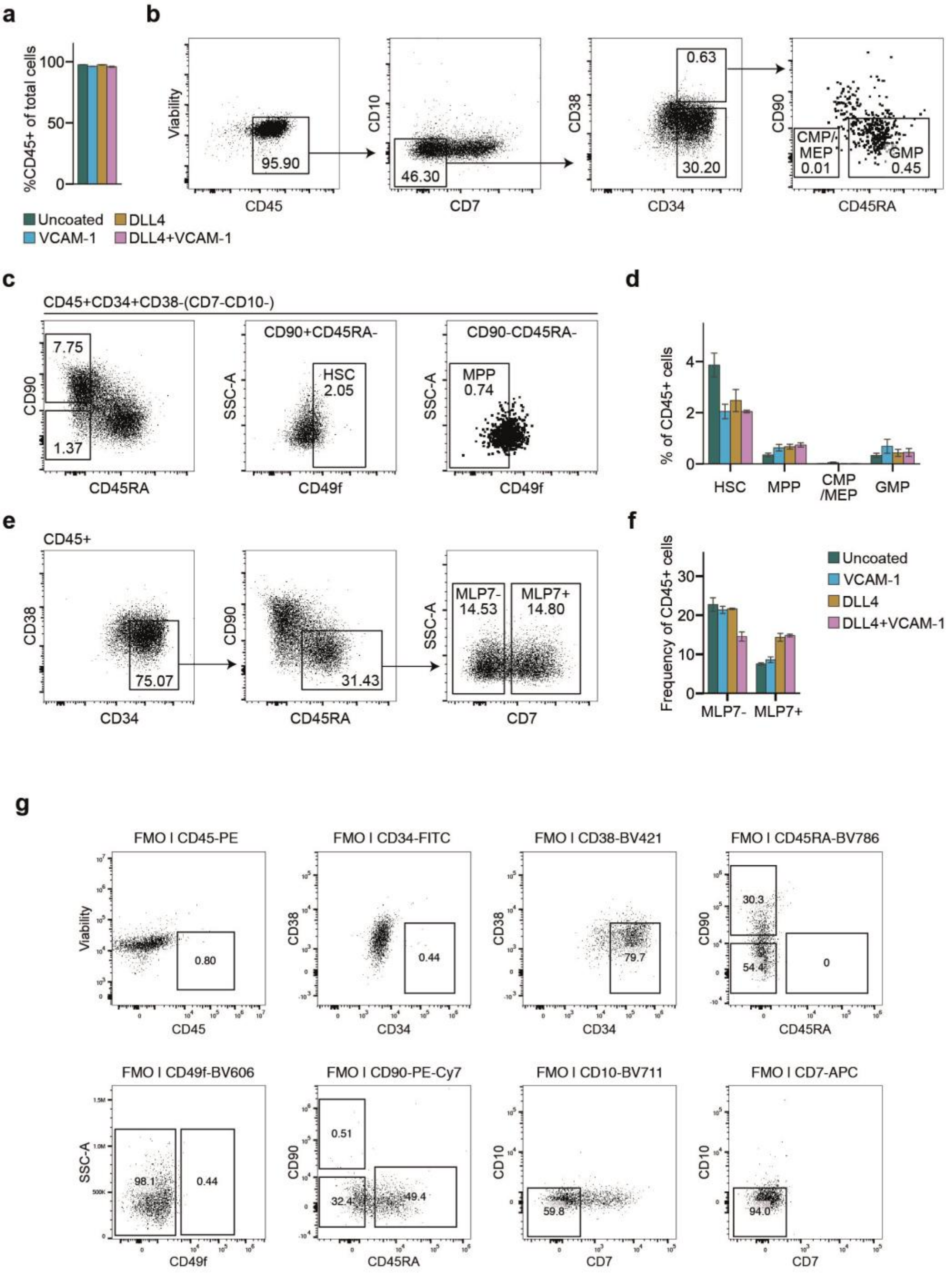

Supplementary Figure 2: Phenotypic characterisation of PSC-derived Hematopoietic Stem and Progenitor Cells. a.) Quantification of CD45+ expression by flow cytometry of non-adherent hematopoietic cells generated after 5 days of EHT (n=3, mean +/- s.d). **b.**) Representative flow cytometry plots and gating strategy to identify common myeloid progenitor/megakaryocyte-erythroid progenitors (CMP/MEP) and granulocyte-monocyte progenitor (GMP). Data shown are from the DLL4 + VCAM1 coating condition and are representative of n=3 differentiation replicates. **c.**) Representative flow cytometry plots and gating strategy to identify hematopoietic stem cells (HSC) and multipotent progenitors (MPP). Data shown are from the DLL4 + VCAM1 coating condition and are representative of

n=3 differentiation replicates. **d.)** Quantification of the flow cytometry data show in (b) and (c) by coating condition. Mean +/- s.d., n=3. **e.**) Representative flow cytometry plots and gating strategy to identify CD7+ and CD7- multilymphoid progenitors (MLP). Data shown are from the DLL4 + VCAM1 coating condition and are representative of n=3 differentiation replicates. **f.)** Quantification of the flow cytometry data show in (e) by coating condition. Mean +/- s.d., n=3. **g.)** Fluorescence-minus-one (FMO) staining controls used to establish gates for the flow cytometry data shown in **b,c** and **e**.

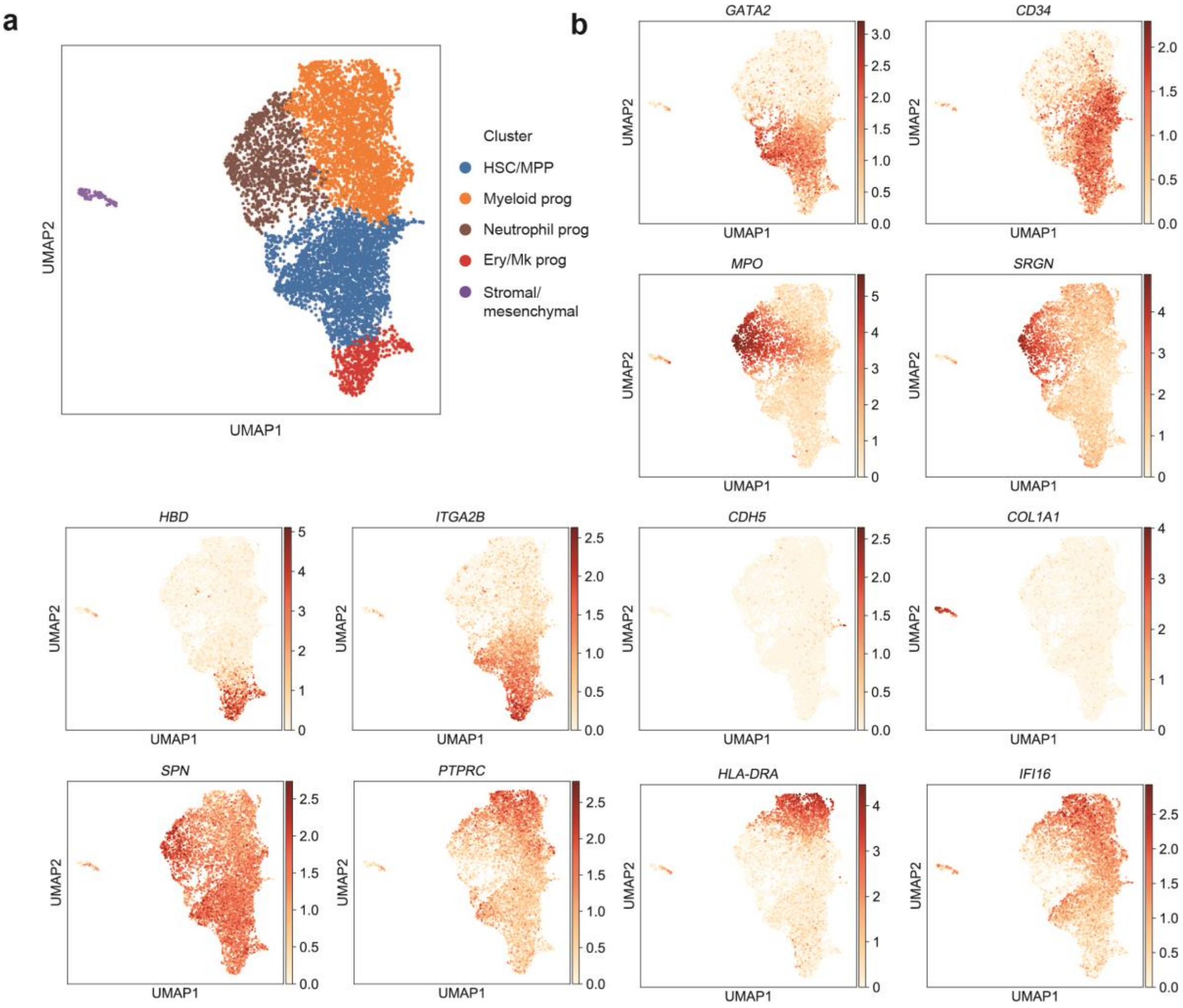

Supplementary Figure 3: Identification of cell types generated during EHT from PSC-derived CD34+ cells. a.) Non-adherent cells that emerged during EHT were collected and analysed by scRNA-sequencing. We performed unsupervised Leiden clustering and identified 5 cell clusters. UMAP projection is coloured by cluster annotation. **b.)** UMAP projection for a selection of marker genes characteristic of each cluster.

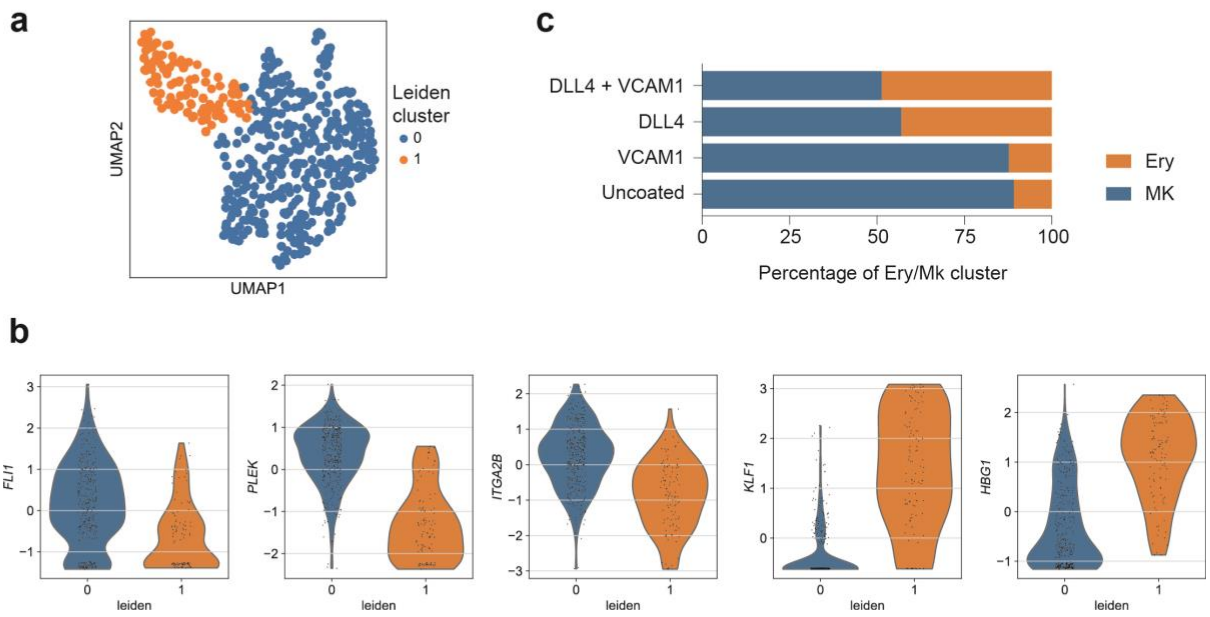

Supplementary Figure 4: Addition of DLL4 during EHT increases the ratio of erythroid to megakaryocyte progenitors. a.) Unsupervised sub-clustering of the Ery/MK progenitors reveal two populations. **b.)** Violin plots of known marker genes demonstrate that cluster 0 displays increased expression of the megakaryocyte-associated genes *FLI1*, *PLEK* and *ITGA2B* while cluster 1 expresses increased levels of the erythroid-associated genes *KLF1* and *HBG1*. **c.)** Clusters from (a) were annotated based on marker gene expression in (b) and the relative abundance of erythroid (Ery) and megakaryocyte (MK) progenitor sub-clusters within the Ery/MK population are plotted for each coating condition.

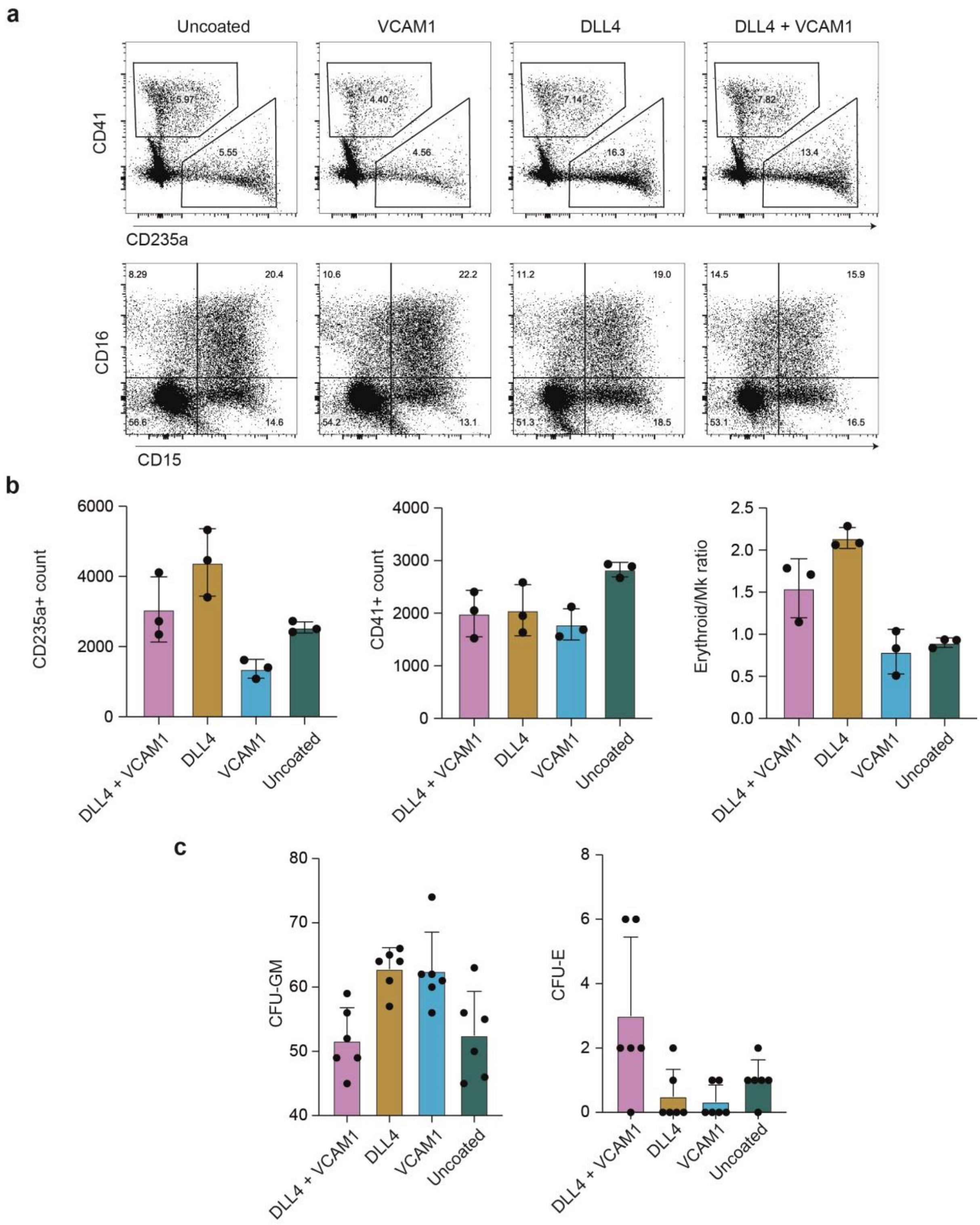

Supplementary Figure 5: Addition of DLL4 during EHT alters erythroid, megakaryocyte and neutrophil output. a.) Representative flow cytometry analysis of extended liquid cultures to mature non-lymphoid hematopoietic progenitors. **b.)** Yields and ratios of CD235+ erythroid cells and CD41+ megakaryocytes determined based on flow cytometry shown in (a). Yields are per well (n = 3, mean +/- s.d). Each well was initially seeded with 2500 PSC-derived cells harvested on day 5 of EHT. 2-way ANOVA revealed that DLL4 significantly affected erythroid/mk ratios (P <0.0001). **c.)** Quantification of colony forming assays of cells harvested at day 5 of EHT from each coating condition (n=6, mean +s.d).

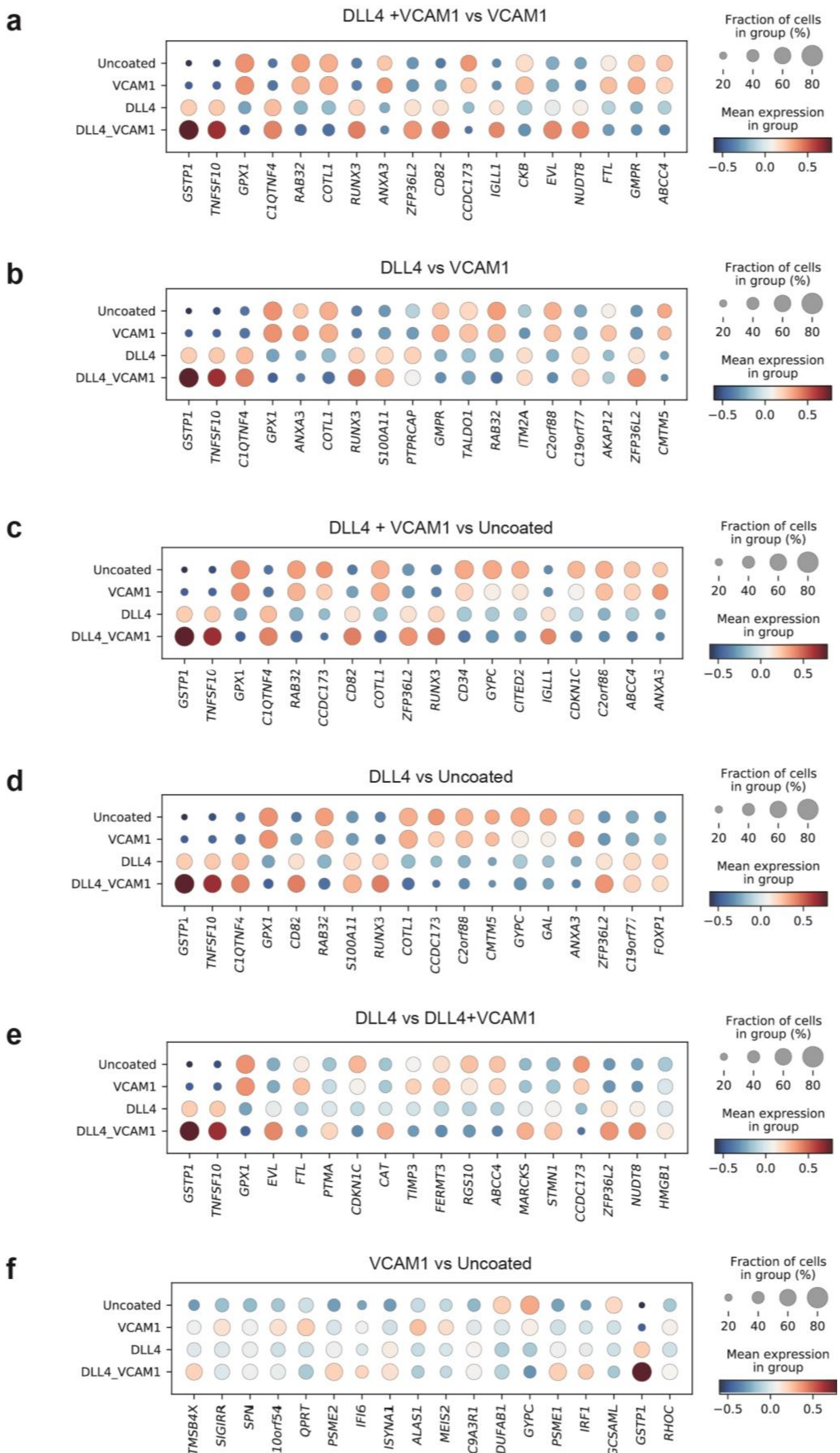
a
DLL4 +VCAM1 vs VCAM1
Uncoated
VCAM1
DLL4
DLL4_VCAM1
GSTP1
TNFSF10
GPX1
C1QTNF4
RAB32
COTL1
RUNX3
ANXA3
ZFP36L2
CD82
CCDC173
IGLL1
CKB
EVL
NUDT8
FTL
GMPR
ABCC4
Fraction of cells in group (%)
20 40 60 80
Mean expression in group
−0.5 0.0 0.5
b
DLL4 vs VCAM1
Uncoated
VCAM1
DLL4
DLL4_VCAM1
GSTP1
TNFSF10
C1QTNF4
GPX1
ANXA3
COTL1
RUNX3
S100A11
PTPRCAP
GMPR
TALDO1
RAB32
ITM2A
C2orf88
C19orf77
AKAP12
ZFP36L2
CMTM5
Fraction of cells in group (%)
20 40 60 80
Mean expression in group
−0.5 0.0 0.5
c
DLL4 + VCAM1 vs Uncoated
Uncoated
VCAM1
DLL4
DLL4_VCAM1
GSTP1
TNFSF10
GPX1
C1QTNF4
RAB32
CCDC173
CD82
COTL1
ZFP36L2
RUNX3
CD34
GYPC
CITED2
IGLL1
CDKN1C
C2orf88
ABCC4
ANXA3
Fraction of cells in group (%)
20 40 60 80
Mean expression in group
−0.5 0.0 0.5
d
DLL4 vs Uncoated
Uncoated
VCAM1
DLL4
DLL4_VCAM1
GSTP1
TNFSF10
C1QTNF4
GPX1
CD82
RAB32
S100A11
RUNX3
COTL1
CCDC173
C2orf88
CMTM5
GYPC
GAL
ANXA3
ZFP36L2
C19orf77
FOXP1
Fraction of cells in group (%)
20 40 60 80
Mean expression in group
−0.5 0.0 0.5
e
DLL4 vs DLL4+VCAM1
Uncoated
VCAM1
DLL4
DLL4_VCAM1
GSTP1
TNFSF10
GPX1
EVL
FTL
PTMA
CDKN1C
CAT
TIMP3
FERMT3
RGS10
ABCC4
MARCKS
STMN1
CCDC173
ZFP36L2
NUDT8
HMGB1
Fraction of cells in group (%)
20 40 60 80
Mean expression in group
−0.5 0.0 0.5
f
VCAM1 vs Uncoated
Uncoated
VCAM1
DLL4
DLL4_VCAM1
TMSB4X
SIGIRR
SPN
C10orf54
QPRT
PSME2
IFI6
ISYNA1
ALAS1
MEIS2
SLC9A3R1
NDUFAB1
GYPC
PSME1
IRF1
GCSAML
GSTP1
RHOC
Fraction of cells in group (%)
20 40 60 80
Mean expression in group
−0.5 0.0 0.5

Supplementary Figure 6: Unbiased exploration of the impact of DLL4 and VCAM1 during EHT on gene expression in PSC derived HSC/MPP. (a-f.) Differential gene expression within HSC/MPP between the specified coating conditions. Pairwise comparisons were performed between each coating condition and all others and the top 18 differentially expressed genes from each comparison by Z-score are depicted (t-test with overestimated variance, Benjamini-Hochberg corrected p-value correction).

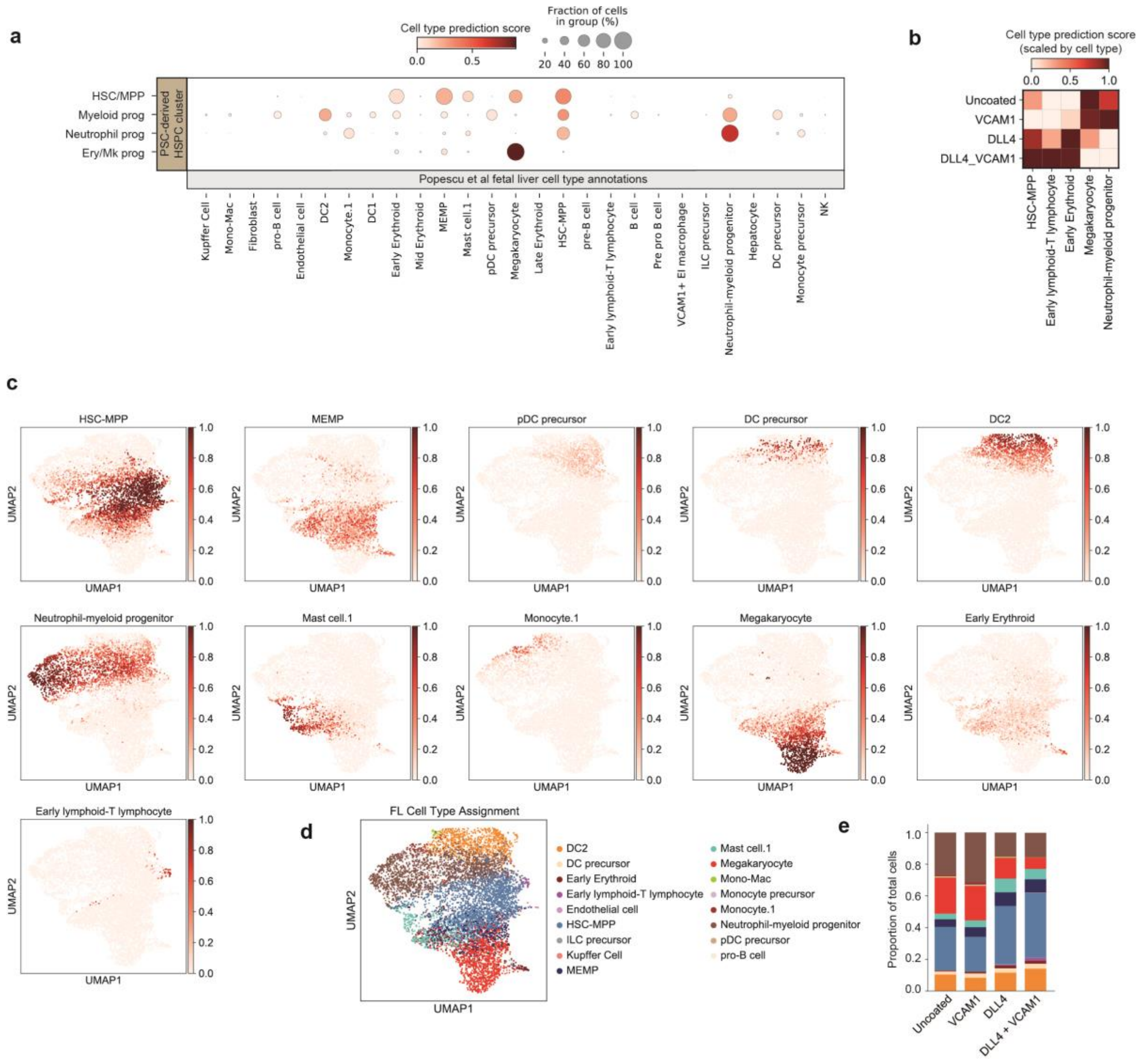

Supplementary Figure 7: PSC-derived HSPC display strong transcriptional correspondence with primary HSPC. a.) scRNA sequencing data from PSC-derived HSPC were integrated with a previously published dataset from primary human fetal liver (FL) from Popescu et al (58). For PSC-derived cells, we calculated a transcriptome-wide cell type prediction score for each labelled cell type in the Popescu et al. (58) dataset using an anchor-based mutual-nearest-neighbours integration method established by Stuart et al (57). Dotplot shows the transcriptome-wide scores for each FL cell type label (columns) broken down by our PSC-derived HSPC clusters (rows). This analysis shows strong agreement between our PSC-derived cells types and their closest corresponding primary cell labels. **b.)** Comparison of a selection of transcriptome-wide FL cell type scores across coating conditions. **c.)** Transcriptome-wide FL cell type prediction scores plotted on UMAP projections of PSC-derived HSPC. **d.)** We classified PSC-derived cells into the FL cell type labels for which they had the highest transcriptome-wide cell type prediction score and plotted these new annotations on the UMAP projection. **e.)** After classifying PSC-derived cells into FL cell type labels, we quantified the frequency of each cell type and plotted them by EHT coating condition.

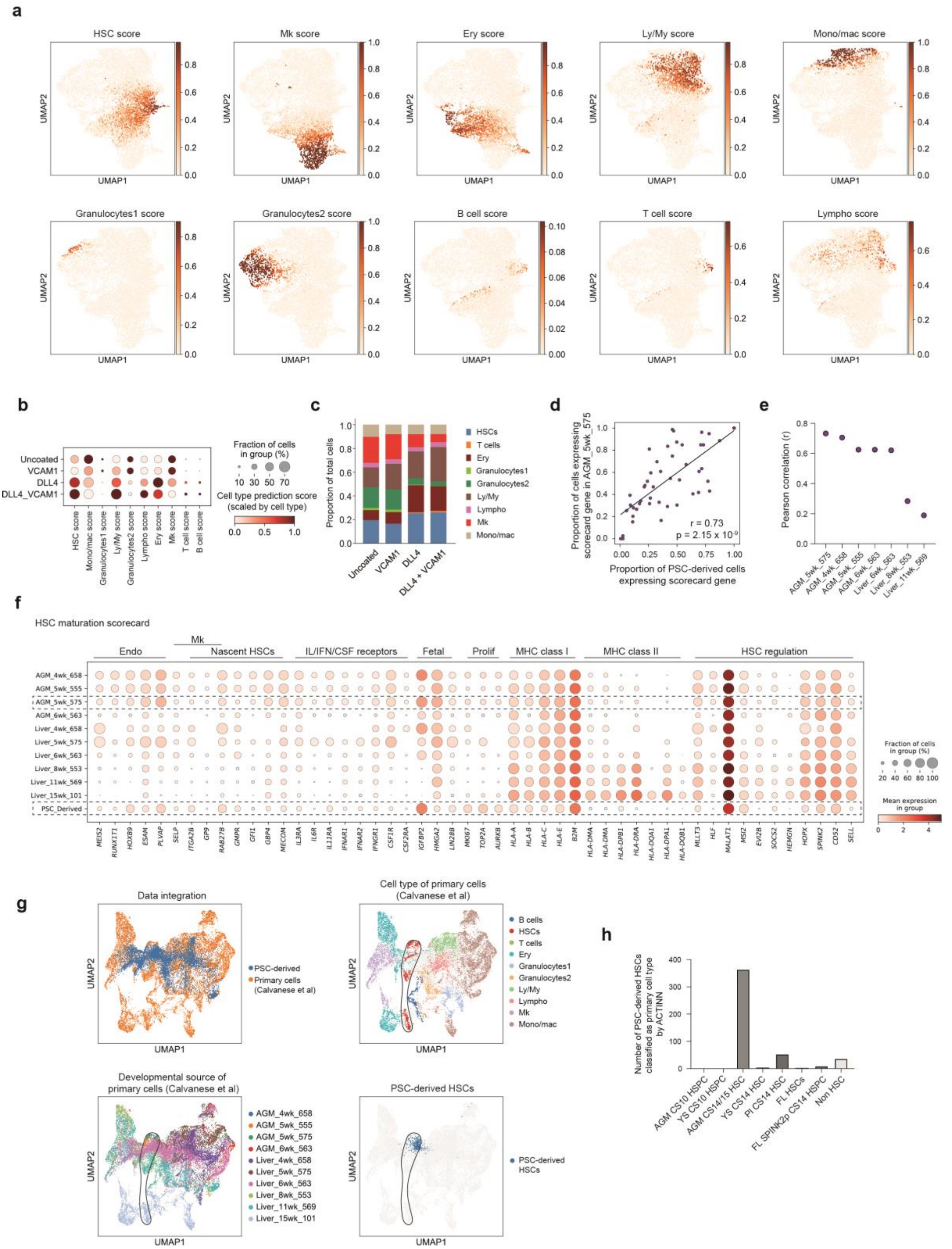

Supplementary Figure 8: PSC-derived HSCs display strong transcriptional correspondence with primary HSCs from the human CS14/15 AGM. a.) scRNA sequencing data from our PSC-derived HSPC were integrated with a recently published dataset from primary human hematopoietic development Calvanese et al. (59) using an anchor-based global integration method (57). For PSC-derived cells, we calculated a

transcriptome-wide cell type prediction score for each labelled cell type in the Calvanese et al. (59) dataset. UMAPs show cell type prediction scores from primary cells plotted on PSC-derived HSPC. **b.)** Dotplot shows the scores for each primary cell type label (columns) broken down by the coating condition used during EHT to generate our PSC-derived HSPCs (rows). **c.)** After classifying PSC-derived cells into primary cell types from Calvanese et al. (59) using a transcriptome-wide anchor-based integration strategy, we quantified the frequency of each classified cell type and plotted them by EHT coating condition. This analysis corroborates the findings noted previously in our study that DLL4 increases HSC and erythroid production while decreasing granulocyte and megakaryocyte (Mk) output. Ly/my= lymphoid/myeloid progenitor, ery = erythroid progenitor. **d,e.)** We compared the transcriptional identity of PSC-derived cells that were classified as HSCs with primary HSCs from different anatomical locations and developmental timepoints by comparing expression of genes from an “HSC maturation scorecard” established by Calvanese et al. (59). In **(d)** We show an example regression comparing PSC-derived HSCs to their most-similar primary counterpart, HSCs from the 5-week AGM. The numbers after each primary cell type label are sample identifiers from Calvanese et al. (59). Note that there are two biological replicates for the 5-week AGM (samples 555 and 575). **f.)** Dotplot showing expression of genes from the HSC maturation score card used for analysis in **(d)** and **(e).** Dashed box highlights our PSC-derived HSCs and their most similar primary counterpart, a sample from the 5-week AGM. **g.)** To further compare our PSC-derived cells to primary cells from Calvanese et al (59), we integrated these two datasets using the Scanpy ‘ingest’ function and plotted them in a UMAP. This analysis revealed that our PSC-derived HSCs occupy a position in the two-dimension projection that overlaps with HSCs from week 5 and 6 AGM and week 6 fetal liver HSCs. **h.)** To provide further unbiased, transcriptome-wide characterisation of our PSC-derived HSCs, we used Automated Cell Type Identification using Neural Networks (ACTINN, 60) to classify our cells into their most similar primary counterparts. We used a training dataset from Calvanese et al (59) that comprises multiple hematopoietic cell types from different developmental times and anatomical locations. This neural network model classified the majority of our PSC-derived HSCs as definitive HSCs from the Carnegie stage (CS) 14/15 AGM. The ‘Non HSC’ category contains all cells classified as hemogenic endothelium, arterial endothelium, erythroid/megakaryocyte/mast progenitors or monocyte/macrophage progenitors. Zero PSC-derived HSC cells were classified as granulocytes, erythroid progenitors, granulocytes, mature monocytes/macrophages, T lymphocytes, B lymphocytes or cord-blood HSCs.

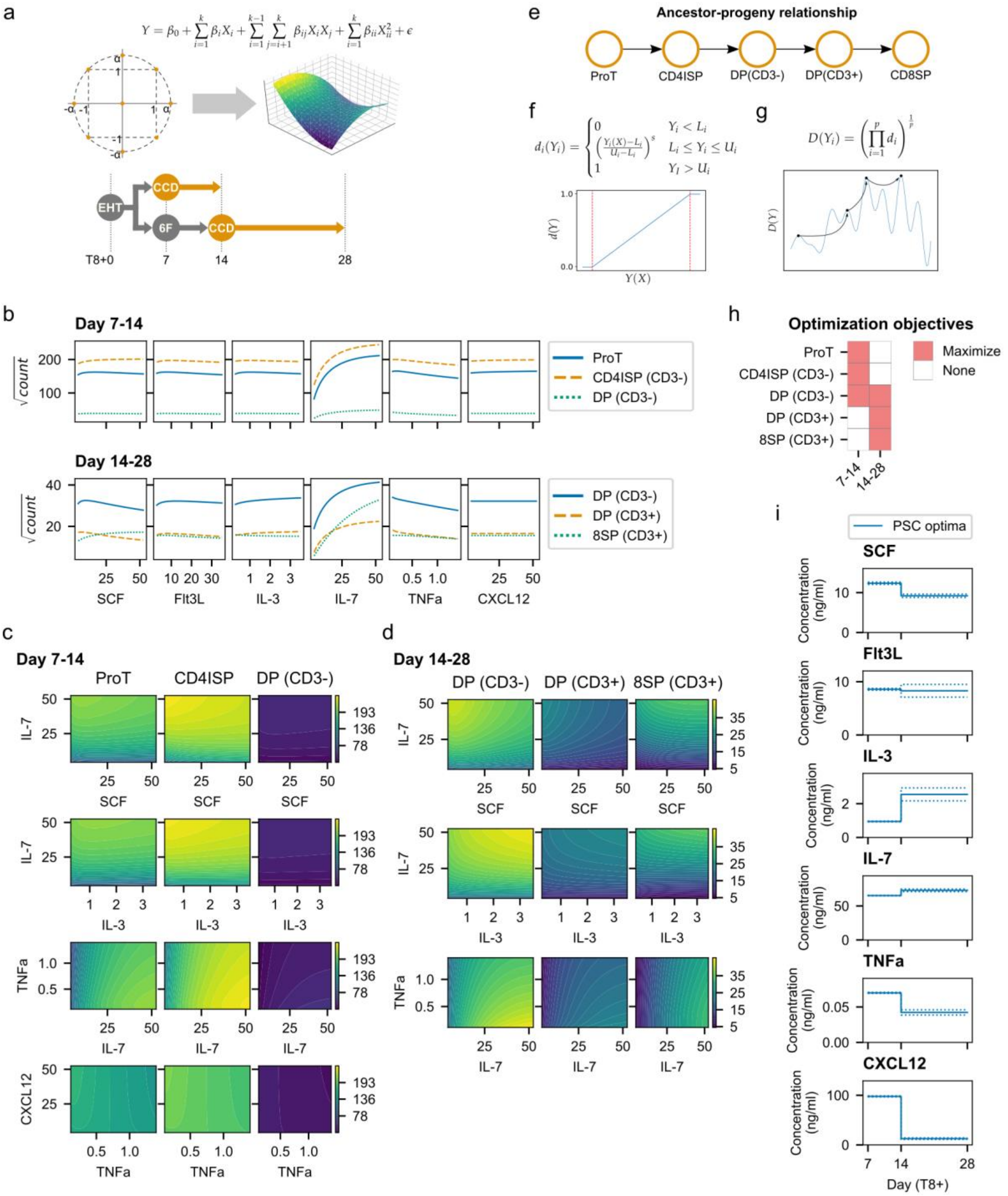

Supplemental Figure 9. Modelling cytokine dose responses and optimization throughout T-cell development. **a.)** A 6-factor orthogonal central composite design (CCD) experiment was performed at two stages of T-cell differentiation (T8+7-14 and T8+14-28). A polynomial equation fit using least-squares regression was used to model the dose response for each population of interest. **b.)** Predicted dose response for each population measured and for each cytokine. **c.)** Significant two-factor interactions between cytokines during day 7-14. **d.)** Significant two-factor interactions between cytokines during day 14-28. **e.)** In order to optimize cytokine concentrations to generate CD8+ T-cells, an ancestor-progeny relationship was assumed, where increasing the number of early phenotypes (ie. proT-cells and CD4ISP) would lead to larger numbers of DP and CD8SP T-cells later. **(f)** For

each population of interest i, a desirability function $d(Y_i)$ was defined which scales the output of each polynomial model $Y_i(X)$ between [0,1]. Cytokine concentrations X that increased Y_i result in a desirability closer to 1 (more desirable) whereas those that decrease Y_i are closer to 0 (less desirable). **g.)** The desirability function for each population was combined using the geometric mean to provide an overall desirability score D which was optimized using the single objective basin-hopping algorithm. **h.)** Objective for each population of interest. Day 7-14 focused on early phenotypes while day 14-28 focused on more mature phenotypes once they were present in culture. **i.)** Predicted optimal cytokines for each stage. 25 random cytokine concentrations were used to initialize the basin-hopping algorithm and the top 5 most desirable solutions were kept. The solid line represents the mean of the top 5 solutions while the dotted line is the standard deviation. A larger standard deviation indicates that optimal solutions were less sensitive to that particular cytokine.

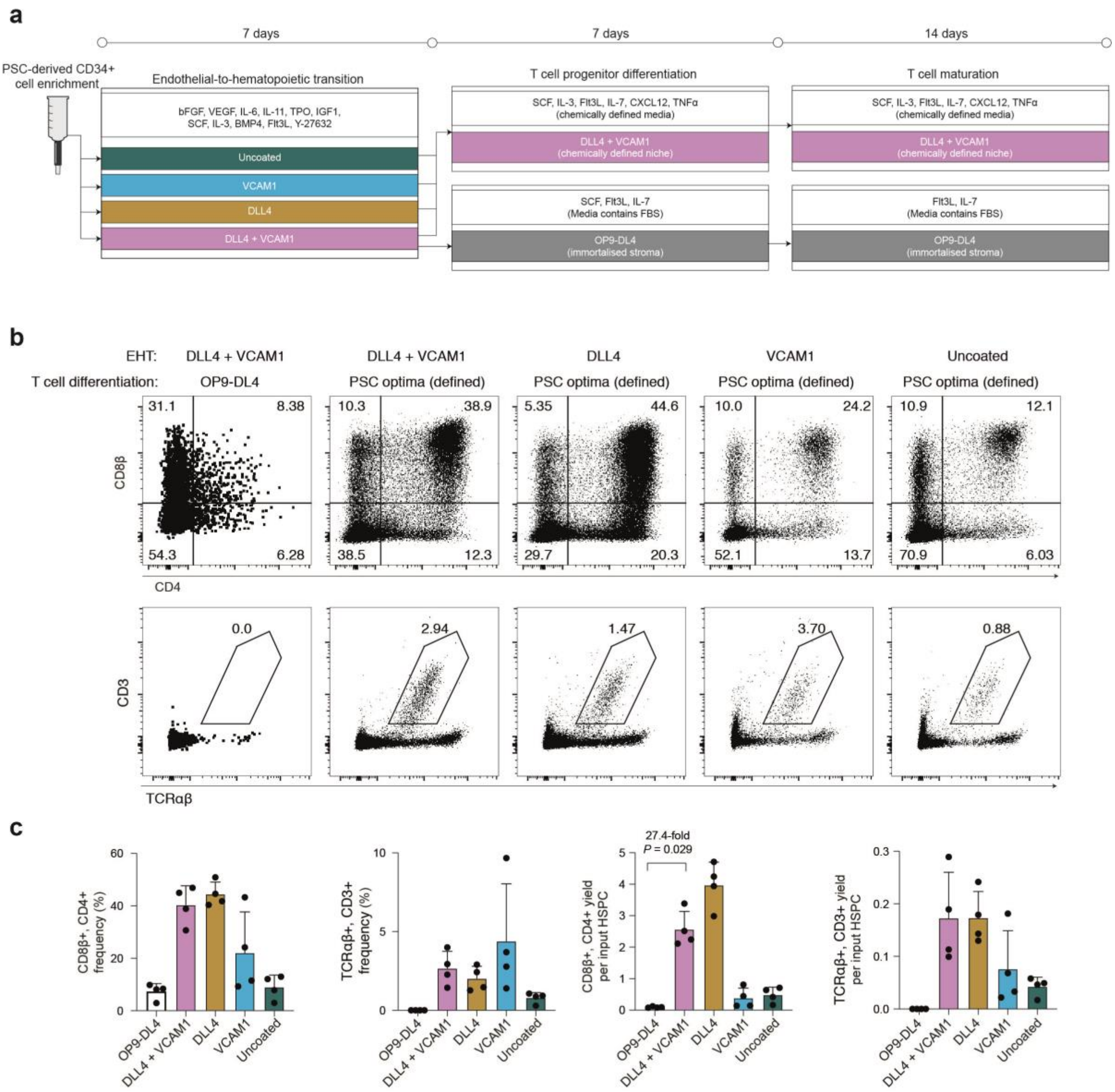

Supplemental Figure 10. An optimised and chemically defined differentiation process improves TCRαβ+ T cell production from PSCs. a.) Experimental design to compare the impact of EHT coating conditions on mature T cell phenotypes as well as to compare optimised defined differentiation conditions to OP9-DL4 stromal co-culture. **b.)** Representative flow cytometry plots for CD4 and CD8β expression (top) as well as CD3 and TCRαβ (bottom) for each differentiation condition. c.) Quantification of the flow cytometry data shown in (b). N = 4 differentiation replicates, mean +/- s.d., P value indicates result of Mann Whitney test.

Supplementary Tables

Supplementary Table 1. Cytokine concentrations tested in dose response experiments (ng/ml).

Cytokine	-2.366	-1	0	1	2.366
SCF	0.77	4.29	15	52.5	290.64
Flt3L	0.52	2.86	10	35	193.76
IL-3	0.05	0.29	1	3.5	19.38
IL-7	0.77	4.29	15	52.5	290.64
TNFa	0.02	0.11	0.4	1.4	7.75
CXCL12	0.77	4.29	15	52.5	290.64

Supplementary Table 2. Regression coefficient estimates and statistics for sqrt[proT] during T8+7-14.

Term	Estimate	Std Error	t Ratio	Prob>|t|
Intercept	162.32027	4.08775	39.71	<.0001
SCF	1.1753401	2.161302	0.54	0.59
Flt3L	-2.297715	2.161302	-1.06	0.295
IL-3	0.3934496	2.161302	0.18	0.8566
IL-7	73.429514	3.19335	22.99	<.0001
TNFa	-10.05826	2.161302	-4.65	<.0001
CXCL12	2.6194807	2.161302	1.21	0.2336
SCF*IL-7	-5.546098	2.511207	-2.21	0.0338
IL-3*IL-7	1.0284591	2.511207	0.41	0.6846
IL-7*TNFa	0.4123762	2.511207	0.16	0.8705
TNFa*CXCL12	-5.542122	2.511207	-2.21	0.034
SCF*SCF	-6.541473	1.84083	-3.55	0.0011
Flt3L*Flt3L	-5.618722	1.84083	-3.05	0.0043
IL-3*IL-3	-5.199209	1.84083	-2.82	0.0078
IL-7*IL-7	-15.27823	1.84083	-8.3	<.0001
TNFa*TNFa	-7.987426	1.84083	-4.34	0.0001
Block[1]	-11.28556	2.675735	-4.22	0.0002
Block[2]	-9.081582	2.71645	-3.34	0.002
IL-7*IL-7*IL-7	-8.902384	1.072155	-8.3	<.0001

Supplementary Table 3. Regression coefficient estimates and statistics for sqrt[CD4ISP] during T8+7-14.

Term	Estimate	Std Error	t Ratio	Prob>|t|
Intercept	197.23105	3.449441	57.18	<.0001
SCF	6.9739421	1.823812	3.82	0.0005
Flt3L	-0.126888	1.823812	-0.07	0.9449
IL-3	-0.552305	1.823812	-0.3	0.7638
IL-7	69.118812	2.694703	25.65	<.0001
TNFa	-8.101824	1.823812	-4.44	<.0001
CXCL12	1.9329364	1.823812	1.06	0.2965
SCF*IL-7	-16.24081	2.119078	-7.66	<.0001
IL-3*IL-7	-4.824599	2.119078	-2.28	0.029
IL-7*TNFa	1.2495945	2.119078	0.59	0.5592
TNFa*CXCL12	-4.13415	2.119078	-1.95	0.0591
SCF*SCF	-2.913235	1.553381	-1.88	0.0691
Flt3L*Flt3L	-5.529862	1.553381	-3.56	0.0011
IL-3*IL-3	-2.913311	1.553381	-1.88	0.0691
IL-7*IL-7	-13.1999	1.553381	-8.5	<.0001
TNFa*TNFa	-5.861526	1.553381	-3.77	0.0006
Block[1]	-9.370574	2.257915	-4.15	0.0002
Block[2]	-6.356163	2.292272	-2.77	0.0088
IL-7*IL-7*IL-7	-8.829736	0.904736	-9.76	<.0001

Supplementary Table 4. Regression coefficient estimates and statistics for sqrt[DP(CD3-)] during T8+7-14.

Term	Estimate	Std Error	t Ratio	Prob>|t|
Intercept	39.03312	1.007009	38.76	<.0001
SCF	0.212637	0.532433	0.4	0.692
Flt3L	-0.549534	0.532433	-1.03	0.3091
IL-3	-0.274043	0.532433	-0.51	0.61
IL-7	14.123472	0.786675	17.95	<.0001
TNFa	-4.646284	0.532433	-8.73	<.0001
CXCL12	-0.044756	0.532433	-0.08	0.9335
SCF*IL-7	-0.312975	0.618631	-0.51	0.6161
IL-3*IL-7	-0.422433	0.618631	-0.68	0.4992
IL-7*TNFa	-2.074962	0.618631	-3.35	0.0019
TNFa*CXCL12	-1.771969	0.618631	-2.86	0.007
SCF*SCF	-1.272841	0.453485	-2.81	0.0081
Flt3L*Flt3L	-1.444519	0.453485	-3.19	0.003
IL-3*IL-3	-0.457725	0.453485	-1.01	0.3197
IL-7*IL-7	-2.377885	0.453485	-5.24	<.0001
TNFa*TNFa	-1.355425	0.453485	-2.99	0.0051
Block[1]	-2.890302	0.659162	-4.38	0.0001
Block[2]	-0.28152	0.669192	-0.42	0.6766
IL-7*IL-7*IL-7	-1.876824	0.264123	-7.11	<.0001

Supplementary Table 5. Regression coefficient estimates and statistics for sqrt[DP(CD3-)] during T8+14-28.

Term	Estimate	Std Error	t Ratio	Prob>|t|
Intercept	32.174743	1.276881	25.2	<.0001
SCF	-1.939846	1.127087	-1.72	0.0936
Flt3L	0.5175565	0.760114	0.68	0.5002
IL-3	1.5683656	0.760114	2.06	0.0461
IL-7	12.935004	1.127087	11.48	<.0001
TNFa	-2.438299	1.127087	-2.16	0.037
SCF*IL-7	-1.920256	0.88782	-2.16	0.0371
IL-3*IL-7	1.9574174	0.88782	2.2	0.0338
IL-7*TNFa	-2.504479	0.88782	-2.82	0.0077
SCF*SCF	-2.756734	0.636598	-4.33	0.0001
Flt3L*Flt3L	-1.398024	0.636598	-2.2	0.0344
IL-7*IL-7	-2.045512	0.636598	-3.21	0.0027
TNFa*TNFa	-1.294971	0.636598	-2.03	0.0491
SCF*SCF*SCF	0.3599793	0.373356	0.96	0.3412
IL-7*IL-7*IL-7	-1.76639	0.373356	-4.73	<.0001
TNFa*TNFa*TNFa	-0.716608	0.373356	-1.92	0.0627

Supplementary Table 6. Regression coefficient estimates and statistics for sqrt[DP(CD3+)] during T8+14-28.

Term	Estimate	Std Error	t Ratio	Prob>|t|
Intercept	16.533849	0.819832	20.17	<.0001
SCF	-2.023788	0.723655	-2.8	0.0081
Flt3L	-0.365552	0.488037	-0.75	0.4586
IL-3	0.9113174	0.488037	1.87	0.0698
IL-7	8.5043609	0.723655	11.75	<.0001
TNFa	-1.488186	0.723655	-2.06	0.0468
SCF*IL-7	-1.923721	0.570032	-3.37	0.0017
IL-3*IL-7	1.1479938	0.570032	2.01	0.0513
IL-7*TNFa	-0.908999	0.570032	-1.59	0.1193
SCF*SCF	-1.377391	0.408733	-3.37	0.0018
Flt3L*Flt3L	-1.109826	0.408733	-2.72	0.01
IL-7*IL-7	-1.641637	0.408733	-4.02	0.0003
TNFa*TNFa	-0.381667	0.408733	-0.93	0.3565
SCF*SCF*SCF	0.1718311	0.239716	0.72	0.478
IL-7*IL-7*IL-7	-1.008564	0.239716	-4.21	0.0002
TNFa*TNFa*TNFa	-0.657095	0.239716	-2.74	0.0094

Supplementary Table 7. Regression coefficient estimates and statistics for log[8SP + 1] during T8+14-28.

Term	Estimate	Std Error	t Ratio	Prob>|t|
Intercept	5.4937311	0.113162	48.55	<.0001
SCF	0.3277836	0.099887	3.28	0.0023
Flt3L	-0.075585	0.067364	-1.12	0.2691
IL-3	-0.050463	0.067364	-0.75	0.4585
IL-7	1.891356	0.099887	18.94	<.0001
TNFa	-0.060797	0.099887	-0.61	0.5465
SCF*IL-7	-0.077879	0.078682	-0.99	0.3287
IL-3*IL-7	0.2352338	0.078682	2.99	0.0049
IL-7*TNFa	-0.001178	0.078682	-0.01	0.9881
SCF*SCF	-0.082605	0.056418	-1.46	0.1516
Flt3L*Flt3L	-0.097429	0.056418	-1.73	0.0925
IL-7*IL-7	-0.244079	0.056418	-4.33	0.0001
TNFa*TNFa	-0.104099	0.056418	-1.85	0.073
SCF*SCF*SCF	-0.047102	0.033088	-1.42	0.163
IL-7*IL-7*IL-7	-0.162448	0.033088	-4.91	<.0001
TNFa*TNFa*TNFa	-0.056402	0.033088	-1.7	0.0967

Supplementary Workbook 1. Key reagents and media recipes used in this study
